# Nicotinamide (niacin) supplement increases lipid metabolism and ROS‐induced energy disruption in triple‐negative breast cancer: potential for drug repositioning as an anti‐tumor agent

**DOI:** 10.1002/1878-0261.13209

**Published:** 2022-03-25

**Authors:** Minsun Jung, Kyung‐Min Lee, Yebin Im, Seung Hyeok Seok, Hyewon Chung, Da Young Kim, Dohyun Han, Cheng Hyun Lee, Eun Hye Hwang, Soo Young Park, Jiwon Koh, Bohyun Kim, Ilias P. Nikas, Hyebin Lee, Daehee Hwang, Han Suk Ryu

**Affiliations:** ^1^ Department of Pathology Seoul National University College of Medicine Seoul Korea; ^2^ Department of Pathology Severance Hospital Yonsei University College of Medicine Seoul Korea; ^3^ Center for Medical Innovation Biomedical Research Institute Seoul National University Hospital Seoul Korea; ^4^ School of Biological Sciences Seoul National University Seoul Korea; ^5^ Department of Microbiology and Immunology and Department of Biomedical Sciences Seoul National University College of Medicine Seoul Korea; ^6^ Proteomics Core Facility Biomedical Research Institute Seoul National University Hospital Seoul Korea; ^7^ Department of Pathology Seoul National University Hospital Seoul Korea; ^8^ 112436 School of Medicine European University Cyprus Nicosia Cyprus; ^9^ 35019 Department of Radiation Oncology Kangbuk Samsung Hospital Sungkyunkwan University School of Medicine Seoul Korea

**Keywords:** metabolism, nicotinamide, organoids, proteogenomics, reactive oxygen species, triple negative breast neoplasms

## Abstract

Metabolic dysregulation is an important hallmark of cancer. Nicotinamide (NAM), a water‐soluble amide form of niacin (vitamin B3), is currently available as a supplement for maintaining general physiologic functions. NAM is a crucial regulator of mitochondrial metabolism and redox reactions. In this study, we aimed to identify the mechanistic link between NAM‐induced metabolic regulation and the therapeutic efficacy of NAM in triple‐negative breast cancer (TNBC). The combined analysis using multiomics systems biology showed that NAM decreased mitochondrial membrane potential and ATP production, but increased the activities of reverse electron transport (RET), fatty acid β‐oxidation and glycerophospholipid/sphingolipid metabolic pathways in TNBC, collectively leading to an increase in the levels of reactive oxygen species (ROS). The increased ROS levels triggered apoptosis and suppressed tumour growth and metastasis of TNBC in both human organoids and xenograft mouse models. Our results showed that NAM treatment leads to cancer cell death in TNBC via mitochondrial dysfunction and activation of ROS by bifurcating metabolic pathways (RET and lipid metabolism); this provides insights into the repositioning of NAM supplement as a next‐generation anti‐metabolic agent for TNBC treatment.

## Graphical abstract

Nicotinamide is a niacin (vitamin B3) supplement. Using multiomics systems biology, we showed that nicotinamide increased the activities of reverse electron transport (RET) and lipid metabolic pathways in triple‐negative breast cancer (TNBC), collectively leading to an increase in reactive oxygen species (ROS) levels. The increased ROS level suppressed tumor growth and metastasis in human organoids and xenograft mouse models.

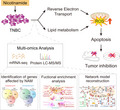

**Abbreviations**
DCFH‐DA: 2'‐7'dichlorofluorescin diacetate
DEGs: differentially expressed genes
DEPs: differentially expressed proteins
FBS: fetal bovine serum
FDRs: false discovery rates
FPKM: fragments per kilobase of transcript per million
G3P: glycerol‐3‐phosphate
GAPDH: glyceraldehyde 3‐phosphate dehydrogenase
GOBPs: gene ontology biological processes
H2O2: hydrogen peroxide
JC‐1: 5,5',6,6'tetrachloro‐1,1',3,3'‐tetraethylbenzimidazol‐carbocyanine iodide
NAC: N‐acetyl cysteine
NAD+: nicotinamide adenine dinucleotide
NAM: nicotinamide
OXPHOS: oxidative phosphorylation
PARP1: poly (ADP‐ribose) polymerase 1
PI: propidium iodide
PS: penicillin/streptomycin
RET: reverse electron transport
ROS: reactive oxygen species
SEM: standard error mean
TNBC: triple‐negative breast cancer
Δψm: mitochondrial membrane potential

## 1 Introduction

Triple‐negative breast cancer (TNBC) is a molecular subtype of breast cancer that shows a dismal clinical behaviour [1]. Unlike other breast cancer subtypes, the conventional cytotoxic chemotherapy still remains the mainstay of systemic treatment for TNBC. Besides, the therapeutic response is expected in just one third of the patients, which encourages the discovery of novel therapeutics [1, 2].

The dysregulation of metabolism is a cancer hallmark that triggers uncontrolled cancer growth [3]. Therefore, the 1 modulation of the metabolic reprogramming has been in the spotlight as a promising option for next‐generation anti‐cancer treatment after immunotherapy [4], Recently, metabolic drugs such as metformin and fluvastatin for diabetes or dyslipidemia have been repurposed as promising anti‐tumour agents showing clinically good outcomes in breast cancer [5, 6]. These findings support reasonable expectations that there might be further candidate compounds having anti‐tumour effects.

Nicotinamide (NAM) is a water‐soluble amide form of niacin (vitamin B3). It is readily taken up by cells and acts as a precursor of coenzyme nicotinamide adenine dinucleotide (NAD^+^). The key mechanism of the supplement is to function as a crucial regulator of mitochondrial metabolism and redox reactions [7]. Several vitamin B complex supplements that contain NAM are now easily available as a dietary ingredient (NIAGEN, ChromaDex Inc., Irvine, CA, USA). Along with the benefits of reversing ageing process and maintaining physiologic functions, anti‐tumour actions of NAM also have recently been addressed in various tumours by regulating SIRT1/2‐, p53‐, AKT‐ and poly (ADP‐Ribose) polymerase 1 (PARP1)‐dependent cascades [8, 9, 10, 11, 12]. Owing to the versatile anti‐cancer actions and cost‐effectiveness of NAM, several clinical trials have been conducted or are in active stages (e.g., NCT03019666 and NCT02416739) concerning the use of NAM in cancer treatment [8, 9, 11, 13].

In the current study, by integrating both transcriptomics and proteomics, we comprehensively investigated metabolic alterations and biologic actions induced by NAM in TNBC at the global levels. The integrative functional enrichment and network analysis of genes and proteins affected by NAM identified a molecular link of NAM‐induced metabolic modulation to the apoptotic death of TNBC cells. NAM suppressed mitochondrial metabolism and increased reactive oxygen species (ROS) by increasing the activity of reverse electron transport (RET) pathway and lipid metabolism, leading to apoptosis in TNBC. Finally, we identified the therapeutic efficacy from the preclinical models of human TNBC organoids and xenograft mice. As a result, we showed strong evidence that NAM suppresses tumour growth and metastasis in TNBC. These findings indicate a promising potential of repositioning NAM supplement as a novel agent targeting cancer metabolism in TNBC.

## 2 Methods

### 2.1 Cell culture and chemicals

BT20 and MDA‐MB‐468 cell lines were obtained from the Korea Cell Line Bank (Seoul, Republic of Korea). MDA‐MB‐231 cells were purchased from the American Type Culture Collection (Manassas, VA, USA). BT20 cells were cultured in RPMI (Gibco, Carlsbad, CA, USA) containing 10% fetal bovine serum (FBS) (Invitrogen, Carlsbad, CA, USA) and 1% penicillin/streptomycin (PS) (Gibco). MDA‐MB‐468 and MDA‐MB‐231 cells were cultured in DMEM (Gibco) containing 10% FBS and 1% PS. Cells were maintained at 37 °C in a humidified atmosphere of 95% air and 5% CO_2_ and periodically screened for *mycoplasma* contamination. All cell lines were confirmed by short tandem repeat DNA profiling tests. N‐acetyl cysteine (NAC) and NAM were purchased from Sigma‐Aldrich (St. Louis, MO, USA). A caspase inhibitor z‐VAD.fmk was purchased from R&D Systems (Minneapolis, MN, USA).

### 2.2 RNA isolation, library preparation and sequencing

Total RNA was isolated from all cell lines (*n* = 3 per cell type) before and 48 h after treatment of 20 mm‐NAM, using TRIzol^®^ RNA Isolation Reagent (Invitrogen), and purified according to the manufacturer’s instructions. The RNA concentration was determined using a NanoDrop ND‐1000 spectrometer (Thermo Fisher, Waltham, MA, USA), and the RNA integrity number for each RNA sample was analyzed using a 2100 Bioanalyzer and the Agilent RNA 6000 Nano Kit (Agilent, Santa Clara, CA, USA). The RNA integrity numbers (RINs) of all samples were higher than 8, which is appropriate for RNA sequencing. The isolated total RNA (1 µg) was utilized to generate cDNA libraries using the TruSeq RNA library kit (Illumina, San Diego, CA, USA), according to the manufacturer’s instructions [14]. The libraries were quantified using qPCR based on the qPCR quantification protocol guide, followed by qualification using an Agilent 2100 Bioanalyzer (Agilent). The sequencing of each library was performed on an Illumina HiSeq 2500 platform and clusters of the cDNA libraries were generated on a TruSeq flow cell and sequenced for 76‐bp paired end reads (2 × 76) with a TruSeq 200 cycle SBS kit (Illumina). Raw data were processed, and base calling was performed using the standard Illumina pipeline (casava ver. 1.8.2 and rta ver. 1.18.64, both provided by Illumina).

For the resulting peptide samples, tandem mass tag 6‐plex labelling was performed according to the manufacturer’s instructions, with modifications. After the labelled peptides were pooled, the sample was separated into 12 fractions using Agilent 1290 bioinert high‐pH reverse‐phase liquid chromatography (HPLC, Agilent) equipped with an analytical column (4.6 × 250 mm, 5 µm). HPLC was performed at a flow rate of 0.8 mL·min^−1^ on a 60‐min gradient using Solvent A (15 mm ammonium hydroxide in water) and solvent B (15 mm ammonium hydroxide in 90% ACN).

### 2.3 Transcriptomic analysis

For the read sequences resulting from the RNA sequencing, the adapter sequences (TruSeq universal and indexed adapters) were removed using the cutadapt software (ver. 2.8) [15]. The resulting reads were then aligned to the human reference genome (GRCh38) using tophat aligner (ver. 2.1.1) with the default options [16]. After the alignment, the mapped reads were counted for gene features (GTF file of GRCh38) using htseq (ver. 0.11.3) [17], and the fragments per kilobase of transcript per million mapped reads (FPKM) were estimated using the gene lengths in Ensembl (GRCh38 Release 99) using cufflinks (ver. 2.2.1).

Next, we identified differentially expressed genes (DEGs) between NAM‐treated samples and non‐treated controls (NAM versus control) in each TNBC cell type using the previously reported statistical method [18]. Briefly, we first selected ‘expressed’ genes as the ones with FPKM values ≥ 1. The log_2_(FPKM+1) was then normalized using the quantile normalization method [19]. For each gene, we calculated the *t*‐statistic values in the comparison of NAM versus control. We estimated an empirical null distribution of the *t*‐statistic values and by performing random permutations of all samples 1000 times. Using the estimated empirical distribution, we computed the adjusted *P*‐value for *t*‐test for each gene. Finally, we identified DEGs as the ones that had *t*‐test *P*‐values < 0.05 and absolute log_2_‐fold‐changes > 0.58 (1.5‐fold) cutoff.

### 2.4 Sample preparation and Liquid Chromatography‐tandem mass spectrometry (LC‐MS/MS)

Three TNBC cell lines (*n* = 3 per cell type) were lysed in SDS‐lysis buffer. Following protein isolation, disulfide bonds were reduced, and sulfhydryl groups were alkylated with 50 mm Iodoacetamide (IAA) solution for 30 min at room temperature in the dark. After the exchange of buffer with 50 mm triethylammonium bicarbonate (TEAB), the protein was digested at 37 °C overnight using the filter‐aided sample preparation procedure, as previously described [20], with a trypsin/LysC mixture at a 100 : 1 protein to protease ratio. LC‐MS/MS analysis was conducted for each fraction of the peptide sample using a Q‐exactive plus mass spectrometry (Thermo Fisher) coupled to an Ultimate 3000 RSLC system (Dionex) and a nanoelectrospray source, as previously described, with modifications [21]. The precursor ions were fragmented with high‐energy collisional dissociation at a normalized collision energy of 32 with a resolution of 35 000 at *m*/*z* 200. The maximum ion injection times for the full scan and MS/MS scan were 20 and 100 ms, respectively. The detailed information for LC‐MS/MS analysis can be found in previous studies [14, 21]. Raw LC‐MS/MS data were uploaded into PRIDE database (PXD005304).

### 2.5 Proteomic data processing and analysis

MS raw files were processed using proteome discoverer 2.1 software (Thermo Fisher). MS/MS spectra were searched with the Human UniProt database (December 2014, 88 657 entries) using the SEQUEST‐HT search engine with forward and reverse protein sequences and common contaminants. Peptides matched to the MS/MS spectra with false discovery rates (FDRs) were determined using the Percolator software package. Reporter ion quantification was performed in the MS2 channel with a 20‐ppm mass tolerance. Detailed search parameters can be found in a previous study [14]. Using the protein abundances, we next identified differentially expressed proteins (DEPs) between NAM‐treated samples and non‐treated controls (NAM versus control) in each TNBC cell type by applying the aforementioned empirical *t*‐test to the normalized protein abundances. After manual inspection of protein abundances between NAM and control, we initially selected DEPs as the proteins with adjusted *P*‐values < 0.2 and absolute log_2_‐fold‐changes > 0.26, 0.28, and 0.33 for BT20, MDA‐MB‐468, and MDA‐MB‐231, respectively. The cut‐off was determined as the mean of 2.5th and 97.5th percentiles of the empirical null distribution for log_2_‐fold‐changes for each TNBC cell type. To further remove false positives, the absolute gaps between NAM and control were calculated for the selected DEPs as the differences between the minimum and maximum abundances in the conditions with high and low mean abundances, respectively. Among the initially selected proteins, we finally selected the final DEPs as the ones with the absolute gap > 0.058, 0.06 and 0.077 for BT20, MDA‐MB‐468, and MDA‐MB‐231, respectively. The cutoff was determined as the mean of 25th and 75th percentiles of the gap distribution in each TNBC cell type.

### 2.6 Gene ontology annotation enrichment analysis

To identify the cellular processes represented by the list of genes or proteins, we performed the enrichment analysis of gene ontology biological processes (GOBPs) for the genes or proteins using david software [22] and then selected the GOBPs with *P*‐value < 0.05 from Expression Analysis Systematic Explorer (EASE) test. To examine the associations among the GOBPs represented by upregulated or downregulated genes/proteins, we next reconstructed a network model where the nodes and edges respectively denote the GOBPs and the associations between the pairs of GOBPs. Two nodes were connected when (a) the number of shared genes (or proteins) commonly involved in the two corresponding GOBPs was ≥3 and (b) the Sørensen–Dice coefficient was > 0.56 for upregulated GOBPs and > 0.32 for downregulated GOBPs. Here, the Sørensen–Dice coefficient between nodes *i* and *j* was determined as 2|*P_i_* ∩ *P_j_* |/(|*P_i_* | + |*P_j_* |), where |*P_i_* | and |*P_j_* | are the numbers of genes/proteins involved in the GOBPs corresponding to nodes *i* and *j*, and |*P_i_* ∩ *P_j_* | is the number of the shared genes/proteins mentioned above, as previously described [23, 24]. We determined the cut‐off (0.56 and 0.32) at the 95th percentile in the coefficient distribution, which was estimated using the coefficients for all possible pairs of nodes with |*P_i_* ∩ *P_j_* | ≥ 3 in the network of upregulated or downregulated GOBPs. For visualization in cytoscape ver. 3.8.2 [25], we used the Community Clustering (GLay) algorithm to the nodes using the clustermaker2 plugin [26], and the resultant node clusters were further grouped into four modules (metabolism, cell proliferation, cell development and immune response).

### 2.7 Reconstruction of molecular network models for DEGs and DEPs

To reconstruct molecular network models, we selected DEGs/DEPs involved in the selected GOBPs. The interactions among the DEGs/DEPs were obtained from metabolic reactions in KEGG pathway database [27, 28, 29]. The resulting networks were visualized using Cytoscape, and the nodes were arranged according to the information in the related KEGG pathways.

### 2.8 Analysis of mRNA‐seq data from TCGA breast cancer cohort

The mRNA‐seq dataset for human breast cancer tissues (TCGA‐BRCA) was obtained from TCGA genomic data commons (GDC) data portal [30] together with clinical information. Among all the samples in the TCGA‐BRCA dataset, we selected 650 breast cancer samples belonging to luminal A, luminal B and TNBC histological subtypes. Note that the HER2‐positive samples were excluded due to the small sample size (29 samples). We downloaded FPKM values of 60 483 genes for 650 samples and then selected the genes with FPKM > 1 in more than 50% of the tumour samples as expressed genes in each subtype. After converting the FPKM values to log_2_‐(FPKM + 1), they were normalized using the aforementioned quantile normalization method. We then applied the empirical *t*‐test mentioned above to the normalized data for the comparison of TNBC (115 samples) versus non‐TNBC (luminal A and luminal B, 535 samples). Finally, we identified DEGs as the ones that had *t*‐test *P*‐values < 0.05 and absolute log_2_‐fold‐changes > 0.18 cut‐off (the mean of 2.5th and 97.5th percentiles of the empirical null distribution).

### 2.9 Measurement of mitochondrial membrane potential

Mitochondrial membrane potential (Δψm) was analyzed by cationic dye 5,5′,6,6′‐tetrachloro‐1,1′,3,3′‐tetraethylbenzimi‐dazolylcarbocyanine iodide (JC‐1, Invitrogen) and MitoTracker Red (CMXRos; Molecular Probes, Carlsbad, CA, USA) staining. Briefly, NAM treated cells were incubated with JC‐1 for 20 min followed by two washes using PBS prior to emission measurements. The emission was measured at 590 and 540 nm after excitation at 480 nm using a multimode microplate reader (Glomax^®^ Explorer; Promega, Madison, WI, USA). For MitoTracker Red analysis, cells were stained with MitoTracker Red (5 μm) for 30 min at 37 °C and analyzed by flow cytometry and processed using the flowjo™ 10 software (BD Biosciences, San Diego, CA, USA). Fluorescent images of the cells were taken under a fluorescent microscope (Leica, Zeiss, Germany).

### 2.10 Oxygen consumption rate (OCR) and real‐time ATP rate analysis

The Mito Stress Test Kit (Cat. 103015–100; Agilent) was used to measure the OCR. The Seahorse XF Real‐Time ATP Rate Assay Kit (Cat. 103592–100; Agilent) was used to detect the ATP production rates of mitochondrial oxidative phosphorylation (OXPHOS). Cells were seeded into XF‐24 cell culture microplates (Seahorse Bioscience, North Billerica, MA, USA) at a density of 40 000–60 000 cells per well and allowed to adhere on a plate overnight. After incubating the cells with NAM for 24 h, OCR and real‐time ATP production rates of mitochondrial OXPHOS were determined and analyzed on the Seahorse Bioscience XF‐24 Extracellular Flux Analyzer (Agilent) according to the manufacturer’s instructions. For the measurement of OCR value, oligomycin, fluoro‐carbonyl cyanide phenylhydrazone (FCCP), and Rotenone/Antimycin A (Rot/AA) were added in order according to the manufacturer’s protocols. For the determination of ATP production rates of mitochondrial OXPHOS and glycolysis, oligomycin and a mix of Rot/AA was added.

### 2.11 Measurement of NAD^+^/NADH ratio

NAD^+^ and NADH levels were measured using a NAD^+^/NADH quantitation kit (Sigma‐Aldrich). Briefly, 5 × 10^5^ cells were lysed. The NAD^+^ level was measured by subtracting the NADH concentration from the total NAD concentration. Data were normalized to total protein content determined by using the BCA protein assay.

### 2.12 Quantification of intracellular ROS

For 2'‐7'dichlorofluorescin diacetate (DCFH‐DA; Sigma‐Aldrich**)** assay, cells were cultured under standard culture conditions up to 80% confluency and different concentrations of NAM was added for 1 h in stressed labelled cells and then incubated at 37 °C with 5 µm DCFH‐DA for 15 min. The fluorescence intensity was measured at 485‐excitation and 530‐emission by fluorimeter as fluorescence units. It was normalized relative to the control as an oxidative stress index. For intracellular hydrogen peroxide (H_2_O_2_) assay, cells were treated with NAM for 24 h in white‐walled clear‐bottom 96‐wells plate and intracellular ROS levels were analyzed by ROS‐Glo™ H_2_O_2_ assay kit (Promega) following the manufacturer’s guidelines. Luminescence was measured using a luminometer (Glomax Explore Multimode Microplate Reader).

### 2.13 Cell viability assay

The procedures described in a previous study were followed [31]. Cells were plated in triplicate (3000 cells per well) and incubated in a medium containing 10% FBS. After 24 h, the complete medium was replaced with the test medium containing the vehicle control and various doses of NAM for 48 h at 37 °C. Cell viability was assessed by measuring the intracellular levels of ATP using the Cell Titer‐Glo luminescent cell viability assay kit (Promega). Luminescence was measured using a luminometer (Glomax Explore Multimode Microplate Reader). To investigate cytotoxicity effects of N‐acetyl cysteine (NAC) or z‐VAD.fmk, cells were seeded into white‐walled 96‐well microplates and treated with NAC or z‐VAD.fmk for 1 h prior to NAM treatment. Following the treatment, an equal volume of Caspase‐Glo 3/7 reagent was added and the luminescence signal was detected using the Glomax‐Multi Detection System (Promega).

### 2.14 Flow cytometry analysis

The procedures described in a previous study were followed [31]. Cell apoptosis assay was performed using the annexin V‐FITC/propidium iodide (PI) apoptosis detection kit (BD Biosciences). Briefly, cells were collected, washed twice with PBS, and then suspended in 300 µL of binding buffer. Annexin V solution (5 µL) was added to the cell suspension and incubated for 15 min in the dark at room temperature. Subsequently, 200 µL of binding buffer and 5 µL of PI were added, and the cell suspension was immediately analyzed on a BD FACSCaliber (BD Biosciences). All data were processed using the flowjo™ 10 software.

### 2.15 Quantitative real‐time polymerase chain reaction (qPCR) and qPCR array

Total RNA was isolated from cells using the AccuPrep^®^ Universal RNA Extraction Kit (Bioneer, Daejeon, Korea) after 24 h of NAM treatment according to the manufacturer's protocol. Genomic DNA was removed by DNase treatment using RNase‐Free‐DNase Set (Qiagen, Hilden, Germany). cDNA was synthesized using AccuPower^®^ RocketScript Cycle RT PreMix (Bioneer). All data were analyzed based on the relative quantification, with normalization to glyceraldehyde 3‐phosphate dehydrogenase (GAPDH) expression for the assessment of *ACSL3*, *CPT2*, *CPT1A*, *HADHB* and *ETFDH* genes and to the reference gene *RPL13A* for solute carrier (*SLC*) array. The ΔΔ*C*_T_ value was used to determine the relative fold change. Table S1 summarizes the primers used for qPCR assays.

### 2.16 Western blotting

Cells were collected and homogenized using radioimmunoprecipitation assay (RIPA) lysis buffer (Thermo Fisher) on ice. Subsequently, the cell lysates were centrifuged at 4 °C to isolate the proteins. Proteins were quantified using the bicinchoninic acid protein assay kit (Thermo Fisher). Western blotting was performed using anti‐c‐caspase 3 (CST, Danvers, MA, USA), anti‐PARP antibody (CST) and OXPHOS cocktail, including NDUFBB, SDHB, MTCO1, UQCRC2 and ATP5A (Abcam, Cambridge, UK). Anti‐GAPDH antibody (BD Biosciences) or anti‐HSC70 antibody (Thermo Fisher) was used as the loading control and quantification of blotting bands were assessed by densitometry (imagej lab software, National Institutes of Health, Bethesda, MD, USA).

### 2.17 Orthotopic and metastatic xenograft mouse model

Animal experiments were conducted in accordance with the gudelines of the Institute for Experimental Animals College of Medicine and the Guide for the Care and Use of Laboratory Animals prepared by the Institutional Animal Care and Use Committee of Seoul National University (no. SNU‐171101‐2). To establish a TNBC xenograft model, 7‐weeks‐old female NOD/SCID mice (NOD.CB17‐*Prkdc^scid^*/J, Jackson Laboratories, Bar Harbor, ME, USA) were housed in cages with a constant‐flow air exchange supporting specific pathogen free condition. Single cell suspensions of MDA‐MB‐468 or MDA‐MB‐231‐luc, cultured in RPMI 1640 medium with 10% FBS and 1% PS (Gibco), were orthotopically implanted into the fourth mammary fat pad with 2.5 × 10^6^ cells. After 3 days, NAM (1000 and 2000 mg·kg^−1^) had been administered in drinking water throughout the experiment. Changes in tumour volume (0.5 × length × width^2^) were measured using digital callipers every 2–3 days from the start of NAM treatment. In addition, changes in body weight and amount of drinking water were measured. MDA‐MB‐231‐luc‐bearing mice were injected with luciferin (PerkinElmer, Waltham, MA, USA) at the end of the experiment and then imaged for bioluminescence signals using IVIS 100 systems (PerkinElmer).

Metastatic tumour burden was histopathologically confirmed from the entire lung, which was formalin‐fixed, paraffin‐embedded and sectioned per every 100 μm. The number of metastatic foci, defined by aggregates of over five tumour cells, was counted using hematoxylin‐eosin slides.

### 2.18 TNBC organoid culture and response to NAM treatment

The organoid experiment using human breast cancer tissues was conducted following the Helsinki standards after approval by the Seoul National Hospital Ethics Committee (no. 2108‐108‐1245), and written informed consent was obtained from patients. TNBC tissues were minced and digested in 10 mL of breast cancer organoid medium containing 1 mg·mL^−1^ collagenase (Sigma) on an orbital shaker at 37 °C for 1–2 h. The digested tissue suspension was strained over a 100‐μm filter and 5% FCS was added to the strained suspension before centrifugation at 400 RCF. Erythrocytes were lysed in 2 mL red blood cell lysis buffer (Roche, Basel, Switzerland) for 5 min at room temperature before the addition of 10 mL organoid washing medium and centrifugation at 400 RCF. The pellet was resuspended in BME type 2, (Trevigen, Gaithersburg, MD, USA) and 40 μL drops of BME‐cell suspension were aliquoated into 24‐well culture plates and solidified at 37 °C for 30 min. Upon completed gelation, 400 μL of breast cancer organoid medium was added to plates. The medium was changed every 4 days and organoids were passaged every 2–3 weeks using TrypLE Express (Invitrogen). To test the efficacy of NAM, organoids were harvested, strained < 70 μm, and then split into a 96‐well plate for 3 days. NAM (20, 40, and 80 mm) as well as control was added in each well for 72 h at 37 °C. Cell viability was assessed by measuring the intracellular levels of ATP using the 3D Cell Titer‐Glo luminescent cell viability assay kit (Promega). Luminescence was measured using a luminometer (Glomax Explore Multimode Microplate Reader).

### 2.19 Statistical analysis of *in vitro* and animal data

All data, unless otherwise indicated, are shown as mean ± standard error mean (SEM) and were analyzed using one‐way ANOVA or two‐tailed *t*‐test using prism 8 (GraphPad, San Diego, CA, USA). qPCR data are presented as the mean ± standard deviation. The half maximal inhibitory concentration (IC_50_) dosages of NAM were calculated by Hill's equation using Prism 8. Sample sizes are indicated in the figure legends. *P*‐values of < 0.05 were considered statistically significant.

## 3 Results

### 3.1 NAM induced mitochondrial dysfunctions in TNBC

**Fig. 1:**
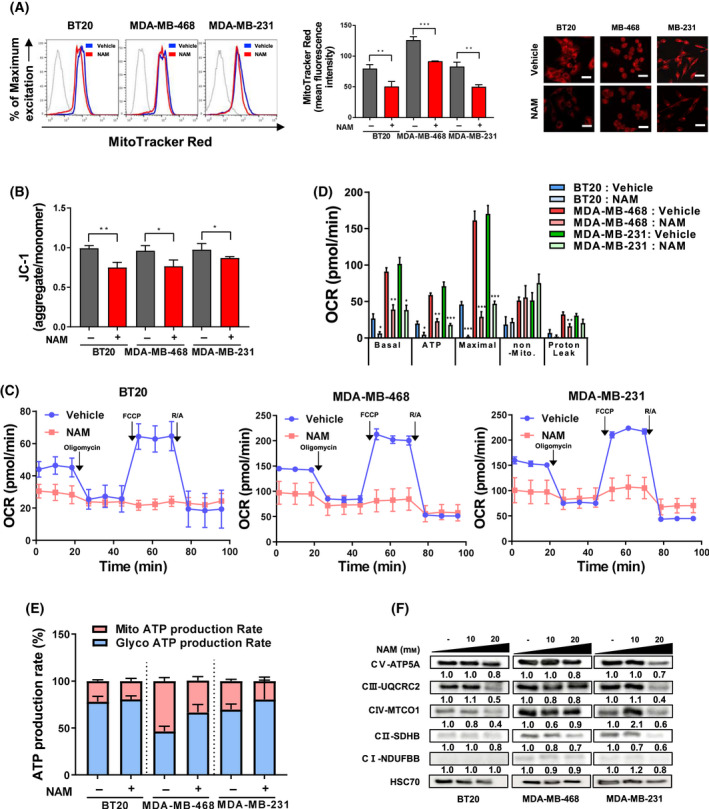
NAM induces mitochondrial dysfunctions in TNBC cells. (A) Mitochondrial membrane potential of triple‐negative breast cancer (TNBC) cells after nicotinamide (NAM) treatment (25 mm, 24 h) was measured using flow cytometry (left) and Mitotracker red staining (250 nm) under confocal microscopy (right; scale bar = 100 μm). (B) JC‐1 (10 nm) fluorescent ratio also indicated that NAM decreased mitochondrial membrane potential. (C) Real‐time mitochondrial oxygen consumption rates (OCRs) after NAM treatment were measured in all TNBC cells by Agilent’s Seahorse XF24 analyzer. (D) Representative graphs of quantification of the basal‐, ATP‐linked‐, maximal‐, nonmitochondrial‐OCR and proton leak after NAM treatment. (E) ATP production rate assay demonstrated alterations in mitochondrial ATP pathway and glycolytic ATP pathway. OCRs and ATP production rate were measured after 24 h of NAM treatment (BT20, 20 mm; MDA‐MB‐468 and MDA‐MB‐231, 40 mm). (F) Western blotting and relative levels of ATP5A, UQCRC2, MTCO1, SDHB and NDUFBB after NAM treatment. The quantification values are normalized to HSC70 as a loading control using the imagej software. Bar graphs are presented in mean ± SEM from triplicate experiment. Comparisons were made using a two‐tailed *t*‐test, *P < 0.05, **P < 0.01, ***P < 0.001. (Abbreviations: JC‐1, 5,5',6,6'tetrachloro‐1,1',3,3'‐tetraethylbenzimidazol‐carbocyanine iodide).

To identify whether NAM is involved in the regulation of metabolism in TNBC, we determined the effect of NAM on Δψm, which is related to the cell’s ability to generate ATP via OXPHOS [32]. CMXRos staining showed that NAM treatment significantly (BT20, *P* = 0.009; MDA‐MB‐468, *P* = 0.0004; MDA‐MB‐231, *P* = 0.002) reduced Δψm in all three TNBC cell types, as evidenced by flow cytometry (left) and confocal microscopy (right) (Fig. 1A). Staining with JC‐1 dye, which forms aggregates at high Δψm but dissolves at low Δψm [13], further confirmed that NAM treatment led to mitochondrial depolarization in all TNBC cell types (BT20, *P* = 0.0043; MDA‐MB‐468, *P* = 0.0184; MDA‐MB‐231, *P* = 0.0368) (Fig. 1B). To examine the dysregulation of ATP production via OXPHOS, we assessed OXPHOS level and real‐time ATP production rate in NAM‐treated cells using Seahorse XF24 Flux Analyzer. The OCR assay showed that NAM profoundly inhibited the mitochondrial OXPHOS process (Fig. 1C), as evidenced by significantly decreased basal respiration (BT20, *P* = 0.0401; MDA‐MB‐468, *P* = 0.0031; MDA‐MB‐231, *P* = 0.004), ATP‐linked respiration (BT20, *P* = 0.0296; MDA‐MB‐468, *P* = 0.0012; MDA‐MB‐231, *P* = 0.0009), maximal respiration (BT20, *P* = 0.0004; MDA‐MB‐468, *P* = 0.0005; MDA‐MB‐231, *P* = 0.0008) and proton leak (MDA‐MB‐468, *P* = 0.0038) capacity 24 h after NAM treatment (Fig. 1D). To further identify the role of NAM in mitochondrial ATP generation, ATP production rates from mitochondria and glycolysis were independently analyzed in TNBC cells after NAM treatment, and a considerable decrease was found in mitochondrial ATP production compared with glycolytic ATP production after NAM treatment (Fig. 1E). Moreover, the levels of mitochondrial complex proteins were reduced by NAM in a dose‐dependent manner (Fig. 1F). Among cocktail proteins, complexes Ⅲ‐Ⅴ showed a noticeable decrease in BT20 and MDA‐MB‐231 cells. Taken together, these results indicated that NAM inhibited OXPHOS and energy metabolism in TNBC cells by reducing mitochondrial membrane potential.

### 3.2 Transcriptomic analysis showed that NAM induced RET‐mediated ROS generation

**Fig. 2:**
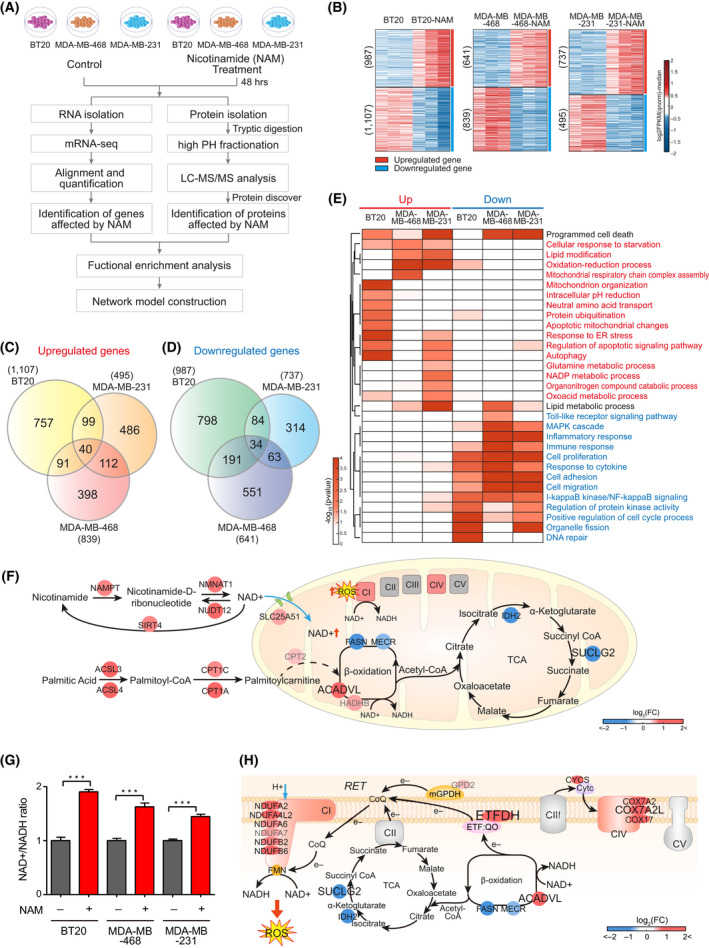
mRNA signatures associated with NAM‐induced mitochondrial dysfunctions. (A) Overall scheme for mRNA‐seq (left) and LC‐MS/MS (right) analyses. (B) Heat maps showing log_2_‐fold‐changes of up‐ (red) and downregulated (blue) genes in BT20 (left), MDA‐MB‐468 (middle) and MDA‐MB‐231 (right) triple‐negative breast cancer (TNBC) cells (colour bar, gradient of log_2_‐fold‐changes of DEGs; numbers of up‐ or downregulated genes are in parenthesis). (C, D) Venn diagrams showing relationships among up‐ (C) and downregulated (D) genes in the three TNBC cells (total = 1983 and 2035, respectively). (E) Heat map showing the significance of cellular processes (gene ontology‐biological processes) being enriched by up‐ (left, red) and downregulated (right, blue) genes in the indicated cell types (colour bar, gradient of –log_10_(*P*); *P* = enrichment *P*‐value from EASE test). (F) Network model describing metabolic reactions altered by nicotinamide (NAM) treatment and the enzymes. Arrows indicate directions of the reactions, light blue arrows denote transport of indicated molecules, and dotted arrows indicate indirect reactions involving intermediate reactions between the nodes. Node or OXPHOS complex colours represent up‐ (red) and downregulation (blue) of the corresponding gene or complex (colour bar, gradient of log_2_‐fold‐changes, NAM versus control). The maximum log_2_‐fold‐changes in the three cell types were chosen for the node colour. Node label size denotes whether the corresponding gene was identified as a DEG in three (large), two (middle) or one (small) of the cell types. Grey node labels denote that the corresponding nodes are non‐DEGs in the three cell types. (G) NAD^+^/NADH ratio in the three TNBC cell types after 10 mm of NAM treatment for 24 h (mean ± SEM from triplicate experiment. Comparisons were made using a two‐tailed *t*‐test, ***P < 0.001). (H) Network model describing RET‐related metabolic reactions affected by NAM treatment. See the legend in (F) for the nodes and edges. (Abbreviations: CI to V, OXPHOS complexes I to V; CoQ, coenzyme Q; Cytc, cytochrome c; e‐, electron; ETF:QO, electron‐transferring‐flavoprotein dehydrogenase; FMN, flavin mononucleotide; mGPDH, mitochondrial glycerol 3‐phosphate dehydrogenase; NAD(H), nicotinamide adenine dinucleotide; RET, reversse electron transport; ROS, reactive oxygen species; TCA, tricarboxylic acid cycle).

As NAM was proven to regulate mitochondrial energy metabolism in TNBC cells, we performed mRNA‐seq analysis to systematically explore the molecular pathways modulated by NAM and how these pathways affect mitochondrial functions (Fig. 2A). In the mRNA‐seq data, we first identified the total number of 1983 upregulated and 2035 downregulated genes in NAM‐treated cells, compared to non‐treated controls, in the three cell types: 2194 DEGs (987 upregulated and 1107 downregulated) in BT20, 1480 DEGs (641 upregulated and 839 downregulated) in MDA‐MB‐468 and 1232 DEGs (737 upregulated and 495 downregulated) in MDA‐MB‐231 (Fig. 2B and Table S2). Among the DEGs, only 40 upregulated and 34 downregulated genes (0.020% and 0.017% of total 1983 upregulated and 2035 downregulated genes, respectively) overlapped in all the types TNBC cells, whereas most DEGs showed cell type‐specific differential expression (Fig. 2C,D).

We next investigated whether cellular processes represented by DEGs in the individual cell types were common across the three types of TNBC cells, despite the low overlapping of DEGs at the molecular level. We identified GOBPs enriched by the DEGs in each cell type and examined whether the enriched GOBPs were shared among the three types of TNBC cells. The upregulated genes in the individual cell types were mainly associated with the processes related to cell death (programmed cell death, autophagy and apoptotic mitochondrial changes) and mitochondria (organization, pH reduction/redox/NADP metabolism, response to starvation, respiratory chain and lipid/oxoacid metabolism) (Fig. 2E, ‘Up’ panel). Interestingly, lipid metabolism and amino acid transport/metabolism (glutamate metabolic process) were also upregulated in one or two TNBC cell types. Among them, cellular responses to starvation and cell death were consistently upregulated in all the three TNBC cell types, reiterating NAM‐induced apoptosis as previously reported [14]. On the other hand, the processes related to immune response (NF‐kB and mitogen‐activated protein kinase signalling, cell adhesion/migration, and response to cytokine) and cell proliferation were downregulated consistently in all the three TNBC cell types (Fig. 2E, ‘Down’ panel).

To investigate how the mitochondrial processes affected by NAM are collectively linked to mitochondrial dysfunction, we built a network model describing the interactions among the DEGs involved in the aforementioned mitochondria‐related processes (NADP metabolism, lipid modification, mitochondrion organization, and respiratory chain complex) from the three cell types of TNBC. The network model suggested that NAM was converted to NAD^+^ by *NAMPT* and *NMNAT1* and then transported to the mitochondria via *SLC25A51* transporter (Fig. 2F) [33]. To verify this result, we measured NAD^+^/NADH ratios in NAM‐treated and non‐treated TNBC cells; NAD^+^/NADH ratios were consistently higher in all the three types of NAM‐treated TNBC cells (BT20, *P* = 0.0002; MDA‐MB‐468, *P* = 0.001; MDA‐MB‐231, *P* = 0.0003) (Fig. 2G). In addition, the network model showed *ETFDH* upregulation and the increased levels of enzymes (*NDUFA2/A4L2/A6/A7/B2/B6*) of OXPHOS complex I (CI), suggesting that the RET pathway was triggered to revert NAD^+^ to NADH (Fig. 2H) [34]. Using qPCR, we verified that the upregulation genes involved in fatty acid β‐oxidation, *ACLS3* (BT20, *P* = 0.0004; MDA‐MB‐468, *P* = 0.0009; MDA‐MB‐231, *P* = 0.0007), *CPT2* (BT20, *P* = 0.0003; MDA‐MB‐468, *P* = 0.0005; MDA‐MB‐231, *P* = 0.0008), *CPT1A*(BT20, *P* = 0.0042; MDA‐MB‐468, *P* = 0.0049; MDA‐MB‐231, *P* = 0.0031), *HADHB* (BT20, *P* = 0.0004; MDA‐MB‐468, *P* = 0.0008; MDA‐MB‐231, *P* = 0.0009), and *ETFDH* (BT20, *P* = 0.0304; MDA‐MB‐468, *P* = 0.0029; MDA‐MB‐231, *P* = 0.029), after NAM treatment (Fig. S1). Fatty acid β‐oxidation facilitates over‐reduction of coenzyme Q, which in turn promotes more RET [35]. Moreover, the levels of enzymes (*IDH2* and *SUCLG2*) involved in the TCA cycle decreased in connection with the electron flux towards RET (Fig. 2F). As the reduction of NAD^+^ to NADH in OXPHOS CI during RET generates high levels of ROS [34], these findings suggested that RET‐ROS generation might be involved in NAM‐induced mitochondrial damage in TNBC cells.

### 3.3 Proteogenomic analysis identified NAM‐induced alteration of lipid metabolism and apoptosis

NAM‐induced modulation linked to mitochondrial dysfunction may involve transcriptional and post‐transcriptional changes that can be observed at the protein level. To augment protein signatures associated with the effect of NAM, we performed proteomic profiling of all the TNBC cell lines (BT20, MDA‐MB‐468 and MDA‐MB‐231). After protein isolation, tryptic digestion and TMT labelling, each labelled peptide sample was fractionated into 12 fractions by employing the previously reported high‐pH fractionation method [21], and the individual fractions were then analyzed using LC‐MS/MS (Fig. 2A). From the LC‐MS/MS data, peptides were identified using the SEQUEST‐HT search engine with an FDR of 1%, corresponding to 9437 proteins, and the abundance of these proteins was estimated using proteome discoverer 2.1 (Thermo Fisher Waltham, MA, USA).

**Fig. 3:**
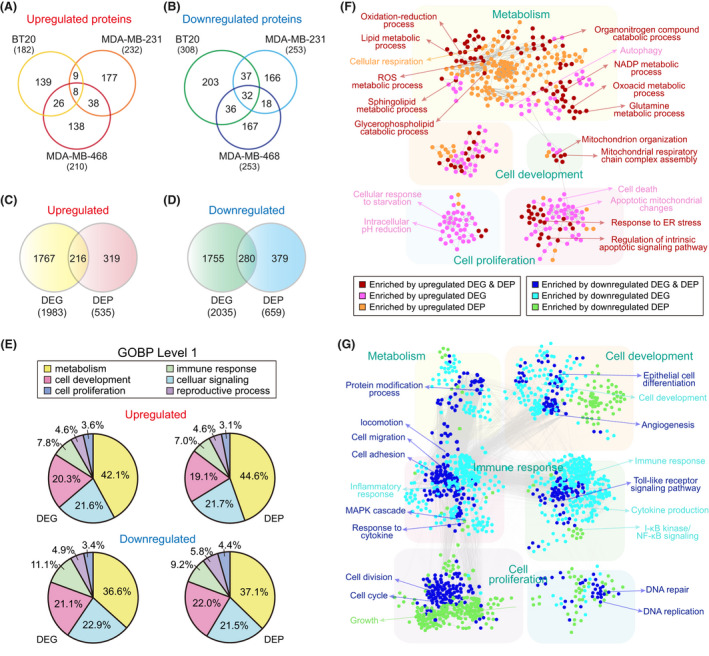
Protein signatures associated with NAM‐induced mitochondrial dysfunctions. (A, B) Venn diagrams showing relationships among upregulated and downregulated proteins in the three triple‐negative breast cancer (TNBC) cells (total = 535 and 659, respectively). (C, D) Relationships between the total up‐ and downregulated genes and proteins. (E) Pie charts showing the percentage of upregulated (top) and downregulated (bottom) genes (left) or proteins (right) involved in the indicated level 1 gene ontology biological processes (GOBPs). (F) GOBP association networks for GOBPs enriched by upregulated genes and/or proteins. (G) GOBP association networks for GOBPs enriched by downregulated genes and/or proteins. GOBP nodes were arranged into four modules (metabolism, cell proliferation and development, and immune response). Edges between GOBP nodes indicate a significant overlap between genes or proteins involved in the corresponding GOBPs.

We identified the total number of 535 upregulated and 659 downregulated proteins in NAM‐treated cells, compared to non‐treated controls in the three cell types: 490 DEPs (182 upregulated and 308 downregulated) in BT20, 463 DEPs (210 upregulated and 253 downregulated) in MDA‐MB‐468 and 485 DEPs (232 upregulated and 253 downregulated) in MDA‐MB‐231 (Table S3). Similar to the DEGs, only a small fraction of the upregulated (8 of 535, 1.50%) and downregulated proteins (32 of 659, 4.86%) were shared across the three TNBC cell lines (Fig. 3A,B). Moreover, among the total DEPs, 216 (40.4% of 535) upregulated proteins and 280 (42.5% of 659) downregulated proteins overlapped with the aforementioned 1983 upregulated and 2035 downregulated DEGs in Fig. 2C,D, respectively, indicating that they were regulated consistently at both the mRNA and protein levels after NAM treatment (Fig. 3C,D).

We then investigated whether cellular processes were commonly represented by the DEPs and DEGs. We first performed GOBP enrichment analysis independently for the following four molecular sets: (1–2) upregulated or downregulated genes and (3–4) upregulated or downregulated proteins. For effective comparison of GOBPs, we examined those level 1 GOBPs that were predominantly enriched by the four molecular sets; we found that the top GOBPs included metabolism, cell development, cell proliferation and immune response for both DEGs and DEPs (Fig. 3E). We therefore categorized level 2–4 GOBPs enriched by the four molecular sets into the level 1 GOBPs and integrated the categorized GOBPs into a GOBP association network, wherein nodes were enriched GOBPs and two nodes were connected when the molecules involved in the corresponding GOBPs were highly overlapping. The GOBP association network for upregulated genes and proteins showed that the processes related to cell death (apoptotic signalling and response to ER stress) and metabolism (mitochondrial organization, redox, cellular respiration/respiratory chain, glycerophospholipid/sphingolipid metabolism and glutamate metabolism) were consistently enriched by both upregulated genes and proteins (Fig. 3F). On the other hand, the GOBP association network for downregulated genes and proteins showed that the processes related to cell development (epithelial cell differentiation and angiogenesis), immune response (cell adhesion/migration, and cytokine production/response to cytokine) and cell cycle (DNA repair and cell division/cycle) were consistently enriched by both downregulated genes and proteins (Fig. 3G).

### 3.4 NAM‐induced metabolic alterations in proteogenomic networks converged on ROS generation and ROS‐mediated apoptosis in TNBC

**Fig. 4:**
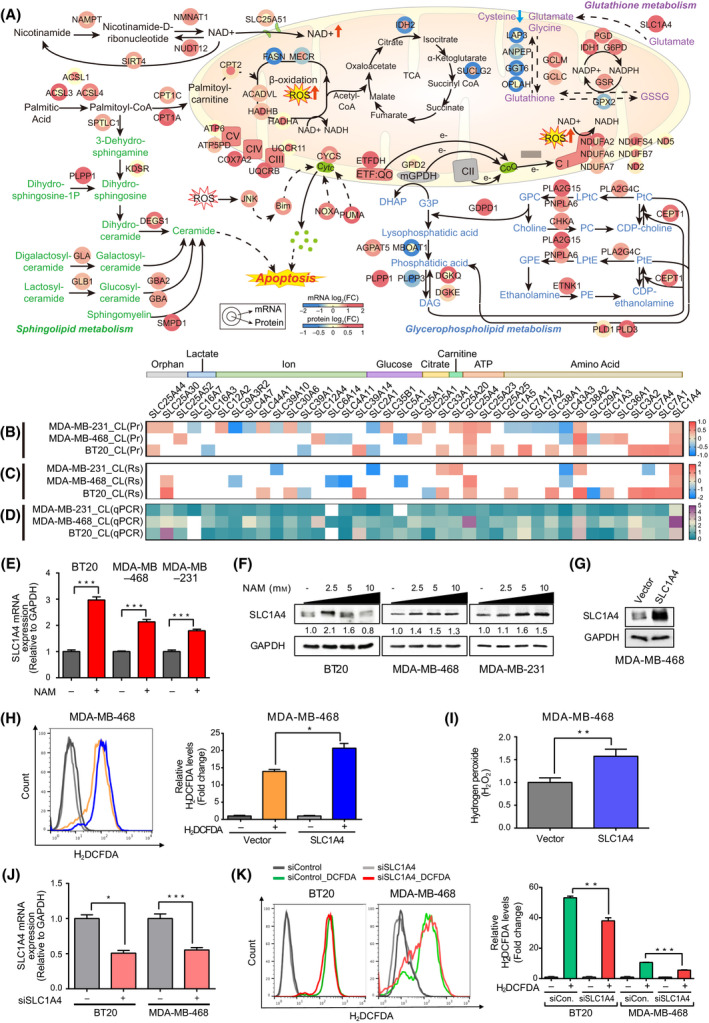
Lipid and glutamate metabolic alterations converge on ROS generation in TNBC. (A) Overall network model describing metabolic reactions that lead to cancer cell apoptosis after nicotinamide (NAM) treatment. Solid and dotted arrows indicate the direct and indirect reactions, respectively. Colour bar indicates the gradient of log_2_‐fold‐changes with respect to the comparison of NAM versus control. Unlike the network models in Fig. 2, the node label size was not used to indicate the number of cell types where the corresponding nodes were upregulated because this network shows the changes of both mRNA and protein levels. (B) Alterations of *SLC* family genes after NAM treatment in proteomic data. (C) Alterations of *SLC* family genes after NAM treatment in RNA‐seq data. (D) Validation of *SLC* family genes using quantitative polymerase chain reaction (qPCR) array. (E) *SLC1A4* mRNA expression after 20 mm of NAM treatment for 24 h. (F) SLC1A4 protein levels after treatment with various concentrations of NAM for 12 h in all TNBC cell lines. (G) Induction of *SLC1A4* gene in MDA‐MB‐468 cells increased the level of SLC1A4 protein. (H) Intracellular reactive oxygen species (ROS) increased by *SLC1A4* overexpression in MDA‐MB‐468 cells by flow cytometry (left) and mean fluorescence intensity (MFI) analysis (right). (I) Cytoplasmic H_2_O_2_ was increased by *SLC1A4* overexpression in MDA‐MB‐468 cells. (J) Selective inhibition of *SLC1A4* by siRNA in BT20 and MDA‐MB‐468 cells decreased *SLC1A4* mRNA. (K) Intracellular ROS was decreased by *SLC1A4* knockdown in BT20 and MDA‐MB‐468 cells by flow cytometry (left) and MFI analysis (right). Bar graphs are presented in mean ± SEM (ROS) or mean ± SD (mRNA) from triplicate experiment (a two‐tailed *t*‐test, *P < 0.05, **P < 0.01, ***P < 0.001). (Abbreviations: CI to V, OXPHOS complexes I to V; CoQ, coenzyme Q; Cytc, cytochrome c; DAG, diacylglycerol; DHAP, dihydroxyacetone phosphate; e‐, electron; ETF:QO, electron‐transferring‐flavoprotein dehydrogenase; G3P, glycerol 3‐phosphate; GPC, glycerophosphocholine; GPE, glycerophosphoethanolamine; GSSG, glutathione disulfide; LPtC, lysophosphatidylcholine; LPtE, lysophosphatidylethanolamine; mGPDH, mitochondrial glycerol 3‐phosphate dehydrogenase; PC, phosphatidylcholine; PE, phosphatidylethanolamine; PtC, phosphatidylcholine; PtE, phosphatidylethanolamine).

In addition to the NAM‐induced NAD^+^‐RET pathway and fatty acid β‐oxidation, the cellular processes enriched by the upregulated genes and proteins included glycerophospholipid/sphingolipid and glutamate metabolism. Several studies have reported that NAM maintains *de*‐*novo* lipogenesis [36] and glycerophospholipid/sphingolipid metabolic processes, which are linked to RET and apoptosis [37, 38, 39]. To determine the cumulative effects of these metabolic processes on NAM‐induced mitochondrial dysfunction and cellular apoptosis, we extended the mRNA network model and include glycerophospholipid/sphingolipid and glutamate metabolic pathways based on both mRNA and protein data (Fig. 4A). The extended network model showed that NAM increased the mRNA and/or protein levels of the enzymes in the glycerophospholipid metabolic pathway to increase the level of glycerol‐3‐phosphate (G3P). Together with the upregulated *ETFDH*, the increased G3P can further enhance the RET pathway and generate RET‐ROS (Fig. 4A, bottom right). Moreover, NAM increased the mRNA and/or protein levels of the enzymes involved in sphingolipid metabolism by increasing the amount of ceramide, which can enhance apoptosis and ROS‐mediated cell death (Fig. 4A, bottom left). In addition to fatty acid β‐oxidation, these data showed additional lipid metabolic pathways modulated by NAM, which eventually results in mitochondrial dysfunction and apoptosis.

To examine clinical relevance of these findings, we next analyzed TCGA mRNA profiles of tumour tissues from 650 patients (115 TNBC, 373 luminal A and 162 luminal B) with BRCA. We first examined key nodes in the above major processes associated with the activation of ROS (RET and glycerophospholipid/sphingolipid metabolism in Fig. 4A) between TNBC and non‐TNBC (luminal A and B). Note that 29 HER2‐positive patients were excluded from the analysis due to its small sample size. Among the 58 key nodes with mRNA expression levels available in TCGA data, 14 were upregulated in TNBC than in non‐TNBC, whereas 21 were downregulated (Fig. S2A–C). When these upregulated and downregulated genes were mapped into the above network model (Fig. 4A), most of the key pathways (*ETFDH* and *GPD2* in RET; *CPT2* and *HADHB* in fatty acid β‐oxidation; *ACSL3*, *SPTLC1*, *PLPP1*, and *CPT1A* in sphingolipid metabolism; and *GDPD1* and *DGKQ* in glycerophospholipid metabolism) were found to be downregulated in TNBC, as well as apoptosis‐related genes (*BIM* and *PUMA*) (Fig. S2D). These data suggest that the activities of the key apoptosis‐inducing processes upregulated by NAM are generally decreased in TNBC, supporting a potential therapeutic value of NAM in patients with TNBC.

In terms of glutamate metabolism, NAM increased the protein (Fig. 4B) and mRNA (Fig. 4C) levels of *SLC1A4* in all the cell lines, which could increase the intracellular glutamate and ROS levels by decreasing the activity of a ROS scavenger, glutathione (GSH) [40]. Additionally, using qPCR array‐based screening of the *SLC* family genes (Fig. 4D), single gene qPCR (Fig. 4E) and western blotting (Fig. 4F), we confirmed that *SLC1A4* was consistently upregulated after NAM treatment in TNBC cells. In the case of the *SLC1A4*‐associated glutamate metabolism pathway, the network model (Fig. 4A, top right) showed that NAM downregulated GSH‐degrading enzymes (*GGT6*, *GPX2*, *ANPEP*, *LAP3* and *OPLAH*) and upregulated GSH‐producing enzymes (*GCLC*/*M*, *GSR*, *IDH1*, *PGD* and *G6PD*). Considering the NAM‐induced *SLC1A4*‐mediated influx of glutamate, these changes towards replenishing GSH may reflect compensation for the decreased GSH levels in NAM‐treated cells. Moreover, the increased glutamate upon NAM treatment might generate additional ROS via oxidative glutamate toxicity [41, 42]. We next confirmed the role of *SLC1A4* in ROS generation. Overexpression of *SLC1A4* (Fig. 4G) increased the ROS levels in MDA‐MB‐468 cells (DCFH‐DA, *P* = 0.0233; H_2_O_2_, *P* = 0.0058) (Fig. 4H,I), whereas knockdown of *SLC1A4* gene (Fig. 4J) decreased the ROS levels in BT20 (*P* = 0.01) and MDA‐MB‐468 cells (*P* = 0.0008) (Fig. 4K).

### 3.5 Metabolic distress by NAM induced ROS‐mediated apoptotic cell death in TNBC

**Fig. 5:**
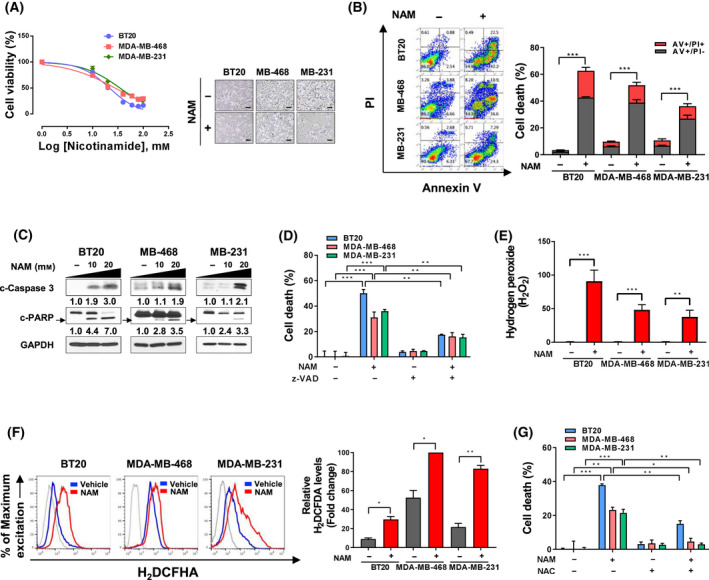
NAM induces ROS‐mediated apoptosis in TNBC cells. (A) Triple‐negative breast cancer (TNBC) cells were treated with increasing concentrations (10–100 mm) of nicotinamide (NAM) for 48 h (left), and cell viability was determined by CellTiter‐Glo Luminescent Cell Viability assay. Representative phase‐contrast images after NAM treatment (right; scale bar = 100 μm). (B) Flow cytometry images of TNBC cells (left) after 48 h of NAM treatment (BT20, 20 mm; MDA‐MB‐468 and MDA‐MB‐231, 40 mm). Apoptosis was determined by Annexin V (AV)/PI‐staining (AV+/PI− and AV+/PI+ cell distribution). The percentage (%) of apoptotic TNBC cells (right) after NAM treatment. (C) Measurement of c‐caspase 3 and c‐poly (ADP‐ribose) polymerase (c‐PARP) (arrow) expression by western blot after NAM treatment for 48 h in TNBC cells. GAPDH served as a loading control. Western blot bands were quantified by densitometry using the imagej software. (D) The cytotoxicity effect of NAM treatment was measured, with or without z‐VAD.fmk pre‐treatment (10 μm; 1 h). Cell death was accessed using CellTiter‐Glo Luminescent Cell Viability assay. (E) Cytoplasmic hydrogen peroxide (H_2_O_2_) was measured by ROS‐Glo H_2_O_2_ assay in the presence or absence of NAM (25 mm). (F) Intracellular reactive oxygen species (ROS) after NAM treatment was assessed using a DCFH‐DA staining (5 μm) by flow cytometry (left) and mean fluorescence intensity (MFI) analysis (right). (G) NAM‐induced cell death altered by 1 mm of N‐acetyl‐L‐cysteine (NAC) pre‐treatment for 2 h. Bar graphs are presented in mean ± SEM from triplicate experiment (a two‐tailed *t*‐test, *P < 0.05, **P < 0.01, ***P < 0.001). (Abbreviations: z‐VAD, carbobenzoxy‐valyl‐alanyl‐aspartyl‐[O‐methyl]‐fluoromethylketone).

To verify the cytotoxicity of NAM and its association with metabolic alterations in TNBC cells that were suggested in the integrative transcriptomic and proteomic analyses, we first treated TNBC cells with dose‐escalated NAM (10–100 mm) and then assessed cell viability. The growth of all TNBC cells was decreased in a dose‐dependent manner after NAM treatment (Fig. 5A). The IC_50_ values of NAM were in the range of 20–35 mm in TNBC cells (Table S4). Flow cytometric analysis also showed that NAM treatment significantly increased the percentage of annexin V‐positive apoptotic cells (annexin V^+^/PI^−^ and annexin V^+^/PI^+^ cells) in all TNBC cell types compared with the non‐treated control (BT20, 3–61%, *P* < 0.0001; MDA‐MB‐468, 9–46%, *P* < 0.0001; and MDA‐MB‐231, 9–34%, *P* = 0.0009) (Fig. 5B). Western blotting of c‐PAPR and c‐caspase 3 corroborated the increased apoptosis of TNBC cells upon NAM treatment (Fig. 5C). In addition, the pretreatment of TNBC cells with z‐VAD.fmk, a pan‐caspase inhibitor, significantly suppressed NAM‐induced apoptosis (z‐VAD/NAM vs NAM, BT20, *P* = 0.0011; MDA‐MB‐468, *P* = 0.0049; MDA‐MB‐231, *P* = 0.0016) (Fig. 5D). These results collectively indicated that NAM inhibited the growth of TNBC cells by activating caspase‐dependent apoptosis.

Because deregulated redox metabolism by NAM may play a pivotal role in triggering ROS‐dependent apoptosis [13], we next investigated NAM‐induced apoptosis mediated by ROS in TNBC cells. In all the NAM‐treated TNBC cell lines, the H_2_O_2_ levels were increased by 40–90 times (BT20, *P* = 0.0007; MDA‐MB‐468, *P* = 0.0004; MDA‐MB‐231, *P* = 0.0028) (Fig. 5E), and the intracellular ROS levels were increased by double or more in the fluorescence dye DCFH‐DA assay (BT20, *P* = 0.0227; MDA‐MB‐468, *P* = 0.0246; MDA‐MB‐231, *P* = 0.0072) (Fig. 5F). In addition, the pretreatment of NAC, an ROS scavenger, followed by the treatment of NAM, showed a significant reduction in NAM‐induced cell death in BT20 (*P* = 0.0044), MDA‐MB‐468 (*P* = 0.01) and MDA‐MB‐231 (*P* = 0.0012) (Fig. 5G). Therefore, these data suggested that the effect of NAM is induced by the upregulation of intracellular ROS levels, which leads to apoptotic cell death in TNBC cells.

### 3.6 NAM supplementation inhibited the growth and metastasis of TNBC in preclinical models

We further examined the tumour‐suppressive effects of NAM supplementation on TNBC by performing *in‐vivo* and *ex‐vivo* preclinical tests. We first adopted xenograft NOD/SCID‐mouse models of two different TNBC cell lines (MDA‐MB‐468 and MDA‐MB‐231‐Luc). It showed that the group received drinking water containing NAM delayed weight gain compared to the control group (Fig. S3A). Measuring the amount of water consumed per mouse demonstrates that the group with drinking water containing NAM consumed 30–50% less than the control group (Fig. S3B). These results suggest that mice were reluctant to drink water containing NAM voluntarily, and the weight gain was delayed by consuming the minimum amount of water for survival.

**Fig. 6:**
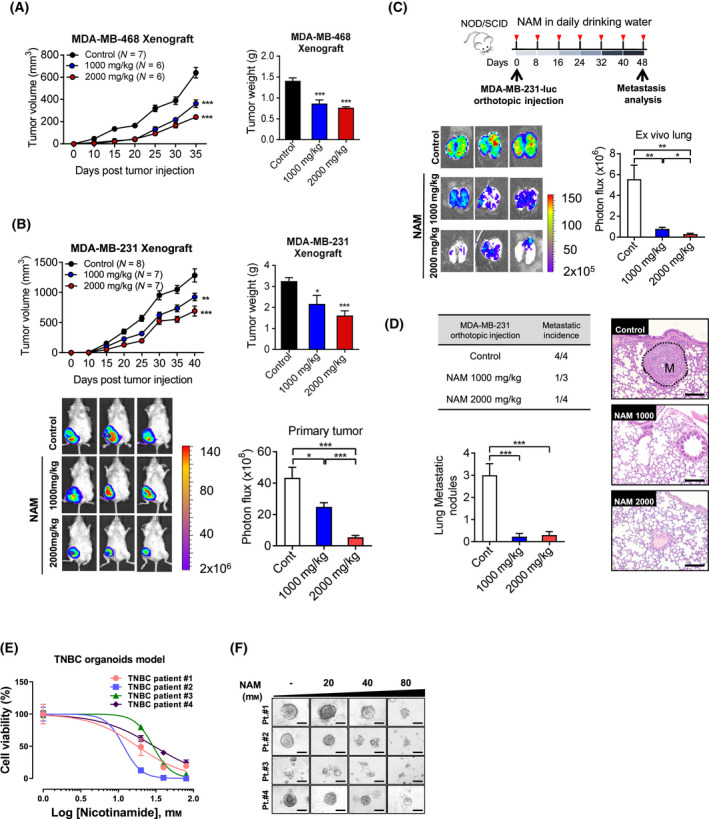
NAM inhibited growth and metastasis of TNBC in animal and organoid models. (A) Growth of orthotopic MDA‐MB‐468 xenograft after NAM (1000 or 2000 mg·kg^−1^) versus vehicle control administered in drinking water. The tumour volume (left) was measured *in vivo* and the weight (right) was measured on day 35 after injection. (B) Growth of orthotopic MDA‐MB‐231‐Luc xenograft after NAM (1000 or 2000 mg·kg^−1^) versus vehicle control administered in drinking water. The tumour volume (top, left) was measured *in vivo* and the weight (top, right) was measured on day 40 after injection. Representative graph shows tumour growth curves on NAM therapy in an established orthotopic xenograft model *in vivo* (top, left). IVIS images (bottom, left) and photon flux (bottom, right) of MDA‐MB‐231‐Luc luciferase signal in mice from indicated treatment groups. (C) Lung metastasis of MDA‐MB‐231‐Luc cells was analyzed 48 days after orthotopic injection with or without NAM in drinking water (top). Representative IVIS images (bottom, left) and photon flux (bottom, right) of luciferase signals in the lung. (D) Metastatic tumour nodules are significantly lower in the NAM‐treated groups compared to the control group (M, metastasis; scale bar = 100 μm). (E) Organoids generated from 4 patients with triple‐negative breast cancer (TNBC) were treated with NAM (20, 40, and 80 mm) for 72 h, and cell death was assessed using 3D CellTiter‐Glo Luminescent Cell Viability. (F) Representative phase‐contrast images depicting 3D spheroid formation of organoids upon NAM treatment (scale bar = 100 μm). Bar graphs are presented in mean ± SEM from triplicate experiment (a two‐tailed *t*‐test, *P < 0.05, **P < 0.01, ***P < 0.001).

Orthotopic tumour burden was significantly lower in NAM‐treated mice than that in non‐treated control in both models, and a dose‐dependent reduction in tumour volume and weight was observed (Fig. 6A,B). In addition, luciferase activity (Fig. 6C) and microscopic examination (Fig. 6D) showed that NAM inhibited pulmonary metastasis of MDA‐MB‐231‐Luc cells 48 days after orthotopic injection. To strengthen our findings that NAM supplementation significantly inhibits the oncogenic properties of TNBC, we next examined the efficacy of NAM using human TNBC organoid models, which better reflect the actual tumour biology than the 2D‐culture of cell lines. Organoids from four TNBC patients were successfully cultured, and viability was measured after NAM treatment for 72 h. Remarkably, NAM treatment showed a strong therapeutic effect by inhibiting the growth of TNBC organoids (Fig. 6E) and disrupting the 3D‐spheroid structures (Fig. 6F). The IC_50_ values of NAM in TNBC organoids were in the range of 12–30 mm (Table S5), which are similar to those found in the TNBC cell lines (Table S4). Taken together, the results showed that NAM supplementation promotes metabolic distress and growth inhibition in TNBC and might be considered a novel anti‐metabolic agent.

## 4 Discussion

Metabolic reprogramming by transforming and building biomass is a general characteristic of tumours to obtain energy so that tumour cells can sustain proliferation and survival in a nutrient‐deprived environment [43, 44]. TNBC is characterized by its dependence on non‐canonical *de‐novo* fatty acid synthesis to fulfil the excessive energy requirement [45]. In a previous xenograft experiment, the inhibition of fatty acid synthase in combination with an anti‐EGFR agent showed synergetic effects against TNBC [46]. The replenishment of NAM has been recently suggested as a new modulator of lipid metabolism in animal models [47]. These findings provided a new strategy for modulating lipid metabolism for TNBC treatment.

In this study, we showed NAM‐induced metabolic disruption in TNBC and the potential role of NAM supplementation as a novel therapeutic agent for TNBC using the combined analysis of multiomics systems biology, xenograft mouse model and patient‐derived organoids. In our network modelling analysis, the perturbation of lipid metabolism was prominent after NAM treatment. Interestingly, the altered lipogenic pathways including fatty acid β‐oxidation and glycerophospholipid and sphingolipid metabolism converged into apoptosis in TNBC by facilitating ROS generation. Significant upregulation of fatty acid β‐oxidation was observed after NAM treatment, which is concordant with a previous report wherein the over‐reduction of OXPHOS CI and coenzyme Q pool by fatty acid β‐oxidation could shuttle electron sources to RET and stimulate RET‐ROS production [35]. In the glycerophospholipid catabolic pathway, *GDPD1* and *GPD2* (mitochondrial glycerol 3‐phosphate dehydrogenase) were significantly upregulated in NAM‐treated TNBC cells. The reduction of G3P by *GPD2* was previously shown to mediate RET and overproduce RET‐ROS in mitochondrial OXPHOS CI much faster than pyruvate‐ or malate‐dependent ROS production [48]. Along with the glycerophospholipid pathway, *SPTLC1*, *ATF6* and *SMPD1*, the key genes associated with the sphingolipid pathway, were also upregulated by NAM treatment. In this cascade, *SMPD1* upregulation was expected to build up ceramide, which induces ROS generation, lysosomal degradation and cell death [49, 50]. In addition to the support of these lipogenic disturbances, we found that NAM‐induced RET‐ROS accumulation was dependent on dysregulated OXPHOS and NAD^+^/NADH imbalance. Based on these findings, we proposed RET‐induced ROS as a therapeutic mechanism of NAM in TNBC; that is, NAM‐mediated RET‐ROS induces mitochondria‐dependent apoptosis. ROS are important signalling molecules involved in energy metabolic processes and play key roles in redox homeostasis in cancer [51, 52]. Recently, alterations in ROS‐related molecular processes have emerged as the hallmarks of TNBC and provided a basis for targeting ROS‐induced metabolic disruption for TNBC treatment [32, 53, 54]. A reverse flow of electron in mitochondrial CI constitutes a considerable source of ROS production, i.e. RET‐induced ROS, which has been implicated in physiologic conditions, including myoblast differentiation, macrophage immune reaction, oxygen sensing in the carotid body and longevity and in pathologic processes, including ischaemia‐reperfusion injury [34]. In cancer, the role of RET‐induced ROS is still unclear [34, 54]. The tumour microenvironment involves recurrent oxidative stress or ischaemic injury, and the RET process might take place in the environment for the control of ROS homeostasis [54]. In addition, there has been other evidence suggesting constitutive promotion of RET in mitochondria, instead of canonical ATP synthesis, in TNBC cells [54, 55]. Thus, our data indicate a pioneering approach using RET‐ROS in TNBC treatment.

We focussed on cellular pathways upregulated by NAM. However, NAM downregulated cell proliferation, adhesion and migration, as well as immune response. Cell proliferation and adhesion/migration are expected to be decreased given the increased ROS. However, how the decrease of the immune‐related genes/proteins measured from TNBC cells may affect their associated tumour microenvironment is not clear. To explore this aspect. we reconstructed a network model describing interactions among the downregulated genes involved in the immune‐related processes (inflammatory response, TLR/NF‐kB/MAPK signalling, cytokine production, and response to cytokine in Figs 2E and 3G) in NAM‐treated TNBC cells (Fig. S4). The network seems to indicate that the increased ROS from RET and lipid metabolism (Fig. 4A) inactivates PI3K/AKT signalling for survival of TNBC cells. This may result in an initial decrease in cytokine production, which then lessens activation of JAK/STAT and MAPK signalling in TNBC cells. As a result of the collective actions of these decreased signalling pathways, the final decreased cytokine milieu appears to be determined. These decreased cytokines include IL1β and LTB, which reduce the activation of MYD88/TRAF2 signalling and in turn the activation of NF‐kB signalling together with the decreased CD14 (TLR4 co‐receptor). Interestingly, these cytokines act on tumour‐associated macrophage (TAM, CCL2/5 and CSF1), myeloid‐derived suppressive cell (MDSC, CCL2/5 and CXCL1/2/5/8/10) and regulatory T‐cell (Treg, CCL2/5/22 and CXCL10) (Fig. S4, table), which are known to suppress the activation of cytotoxic T‐cells, a central player in anti‐tumour immunity. The decrease of these cytokines suggest that NAM might increase the activation of cytotoxic T‐cells and thus promote an immune surveillance environment.

Along with the cross‐talk between NAM and ROS, our preclinical tests showed that NAM supplementation strongly suppressed tumour growth and metastasis in both the xenograft mice model and the human organoid model. In animal models, NAM significantly suppressed tumour growth and distant metastasis of TNBC to the lungs. In the present study, we treated each mouse with 1000 or 2000 mg·kg^−1^ NAM, and no side effects were observed. In a randomized clinical trial, 1000–2000 mg of NAM per day as a supplement showed potential benefits for improving cardiovascular and other physiological functions with limited side effects [56]. The doses used for treating animals are compatible with 80 or 160 mg·kg^−1^ for humans, which are two to three times higher than those reported previously [56]. However, we targeted highly aggressive mammary carcinoma that might require a higher dose of the drug compared with what is required for maintaining general physiological functions. An additional validation test with a patient‐derived organoid model also showed similar therapeutic efficacy of NAM in TNBC. Although there may be a considerable gap between organoids and the human environment, patient‐derived organoids are an excellent preclinical model that provides a platform for the evaluation of drug response in breast cancer [57, 58, 59, 60]. The limitation of this study is that our preclinical models may not reflect the tumour microenvironment, such as adipose tissue. Adipocytes have been revealed to regulate biological processes, including metabolism, inflammation and even the handling of NAM [61, 62]. Taken together, these findings indicated a promising therapeutic potential of NAM that is easily accessible without prescription as a dietary supplement for TNBC treatment.

## 5 Conclusions

To summarize, our findings showed a conceptual signalling pathway in which NAM leads to bifurcating metabolic alterations (RET and lipogenic pathways) in TNBC and promotes the ROS‐induced apoptotic pathway. Moreover, our combined preclinical analysis with *in‐vivo* and *ex‐vivo* models is the first to show that NAM significantly suppresses tumour growth and metastasis in TNBC. We believe that future studies should include clinical trials of NAM by involving TNBC patients to determine the efficacy of NAM supplementation and optimize the dose of NAM in the clinical setting.

## Conflict of interest

The authors declare no conflict of interest.

## Author contributions

MJ: data curation and writing. K‐ML: investigation and writing. YI: formal analysis and writing. SHS, HC, DYK, DH, CHL, EHH, SYP: investigation. JK and BK: resources. IPN: conceptualization. HL: funding acquisition. DH: formal analysis, writing and supervision. HSR: conceptualization, writing, supervision, project administration and funding acquisition. All authors read and approved the final manuscript.

### Peer review

The peer review history for this article is available at https://publons.com/publon/10.1002/1878‐0261.13209.

## Supporting information


**Fig. S1**. The expression levels of genes involved in fatty acid β‐oxidation.

**Fig. S2**. Relative mRNA expression levels of key network nodes in TNBC patients with respect to non‐TNBC patients in the TCGA breast cancer cohort.

**Fig. S3**. Growth curves and voluntary water consumption in the animal model.

**Fig. S4**. A network model for the decreased immune response in NAM‐treated TNBC cells.

**Table S1**. Primers used for qPCR assay.

**Table S2**. DEGs between NAM‐treated cells and controls.

**Table S3**. DEPs between NAM‐treated cells and controls.

**Table S4**. The IC_50_ value of NAM treatment to TNBC cell lines.

**Table S5**. The IC_50_ value of NAM treatment to TNBC organoids.

## Data Availability

The raw and normalized transcriptomic data were deposited in the Gene Expression Omnibus (GEO) database (GSE172494). Raw LC‐MS/MS data were uploaded into PRIDE database (PXD005304).
