# Supplementary material for: Nicotinamide (niacin) supplement increases lipid metabolism and ROS‐induced energy disruption in triple‐negative breast cancer: potential for drug repositioning as an anti‐tumor agent
Source: Mol Oncol. 2022 Mar 25;16(9):1795–815. doi: 10.1002/1878-0261.13209 (PMC9067146; doi:10.1002/1878-0261.13209)
Supplement: Supplementary file 1 — Fig. S1. The expression levels of genes involved in fatty acid β‐oxidation. [file MOL2-16-1795-s008.pdf]

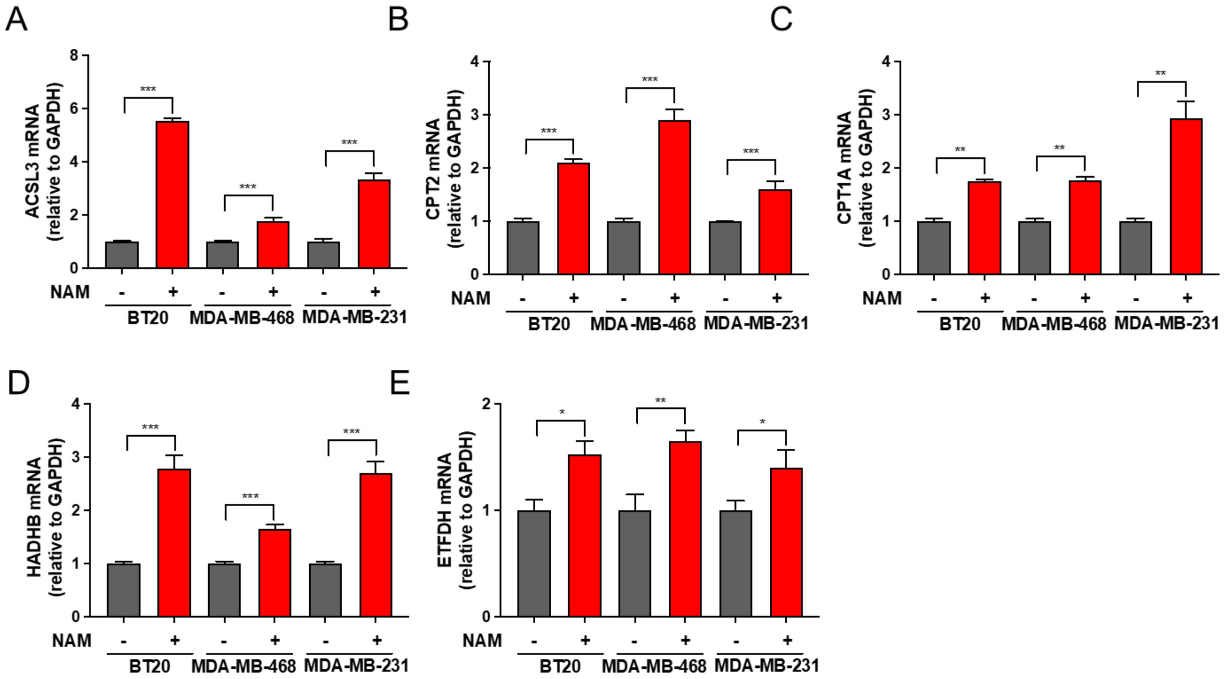

Fig. S1. The expression levels of genes involved in fatty acid β-oxidation. NAM-induced changes (20mM, 24 hours) in expression of *ACSL3* (A), *CPT2* (B), *CPT1A* (C), *HADHB* (D), and *ETFDH* (E) genes by real-time PCR in all three TNBC cell types (two-tailed t-test *$p < 0.05$, **$p < 0.01$, ***$p < 0.001$ from triplicate experiments).
