# Supplementary material for: Nicotinamide (niacin) supplement increases lipid metabolism and ROS‐induced energy disruption in triple‐negative breast cancer: potential for drug repositioning as an anti‐tumor agent
Source: Mol Oncol. 2022 Mar 25;16(9):1795–815. doi: 10.1002/1878-0261.13209 (PMC9067146; doi:10.1002/1878-0261.13209)
Supplement: Supplementary file 2 — Fig. S2. Relative mRNA expression levels of key network nodes in TNBC patients with respect to non‐TNBC patients in the TCGA breast cancer cohort. [file MOL2-16-1795-s005.pdf]

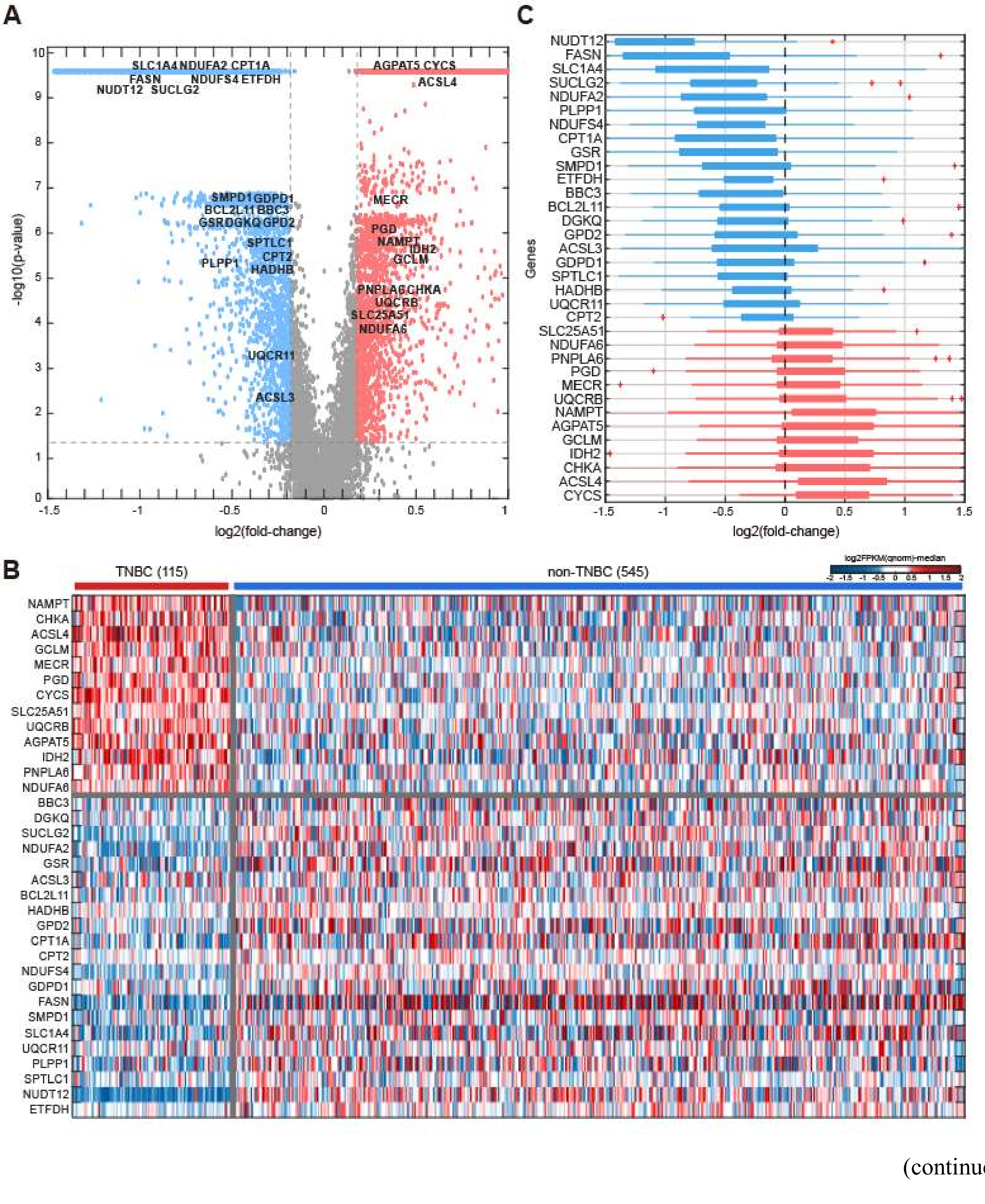

A
C
B
log2(fold-change)
-log10(p-value)
Genes
TNBC (115)
non-TNBC (545)
log2FPKM(qnorm)-median
SLC1A4 NDUFA2 CPT1A
FASN NDUFS4 ETFDH
NUDT12 SUCLG2
AGPAT5 CYCS
ACSL4
SMPD1 GDPD1
BCL2L11 BBC3
GSR DGKQ GPD2
SPTLC1
CPT2
PLPP1
HADHB
UQCR11
ACSL3
MECR
PGD
NAMPT
IDH2
GCLM
PNPLA6 CHKA
UQCRB
SLC25A51
NDUFA6
NUDT12
FASN
SLC1A4
SUCLG2
NDUFA2
PLPP1
NDUFS4
CPT1A
GSR
SMPD1
ETFDH
BBC3
BCL2L11
DGKQ
GPD2
ACSL3
GDPD1
SPTLC1
HADHB
UQCR11
CPT2
SLC25A51
NDUFA6
PNPLA6
PGD
MECR
UQCRB
NAMPT
AGPAT5
GCLM
IDH2
CHKA
ACSL4
CYCS

(continued)

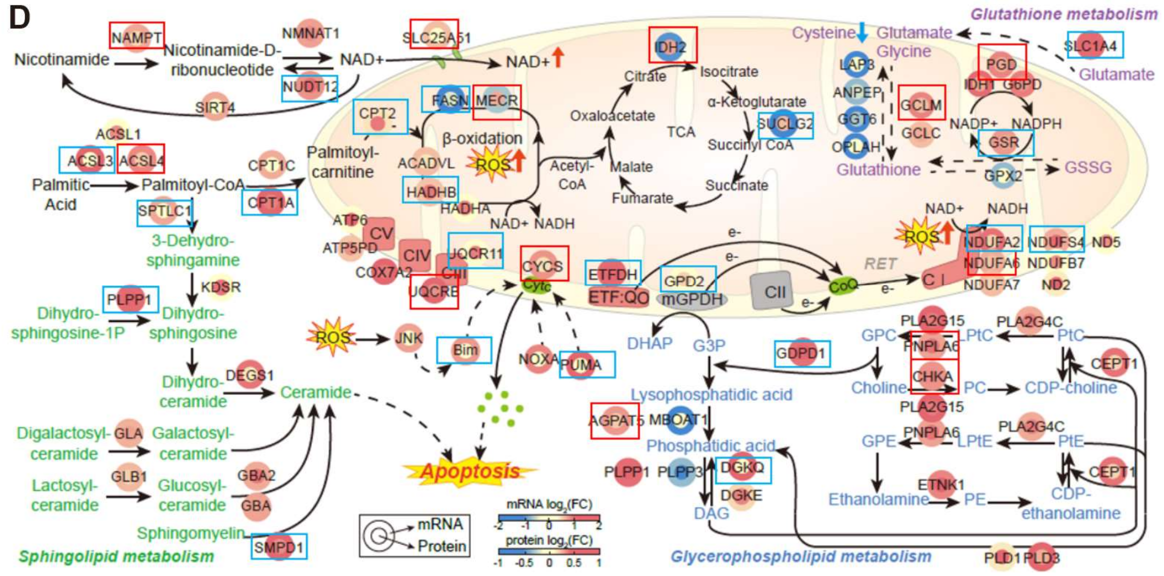

Fig. S2. Relative mRNA expression levels of key network nodes in TNBC patients with respect to non-TNBC patients in the TCGA breast cancer cohort. (A) Volcano plot for the comparison of TNBC versus non-TNBC (TNBC/non-TNBC). X and Y axes represent $\log_2$-fold-changes and the adjusted p-value from the comparison. Dotted lines represent the cutoffs for $\log_2$-fold-change and t-test p-value used in this analysis. Pink and blue dots denote the 3,314 upregulated and 3,362 downregulated genes, respectively. The key nodes were indicated in labels. (B) Heat map showing upregulation (red) and downregulation (blue) of 13 and 21 key nodes in TNBC samples, respectively, compared to in non-TNBC samples. The color bar represents the gradient of $\log_2$-fold-change of mRNA expression levels. (C) Box plots showing expression changes of 13 upregulated and 21 downregulated key nodes in TNBC samples. Outliers were marked with red crosses. (D) The key nodes in the network model showing upregulation (red box) or downregulation (blue box) in TNBC samples.
