# Supplementary material for: Nicotinamide (niacin) supplement increases lipid metabolism and ROS‐induced energy disruption in triple‐negative breast cancer: potential for drug repositioning as an anti‐tumor agent
Source: Mol Oncol. 2022 Mar 25;16(9):1795–815. doi: 10.1002/1878-0261.13209 (PMC9067146; doi:10.1002/1878-0261.13209)
Supplement: Supplementary file 3 — Fig. S3. Growth curves and voluntary water consumption in the animal model. [file MOL2-16-1795-s003.pdf]

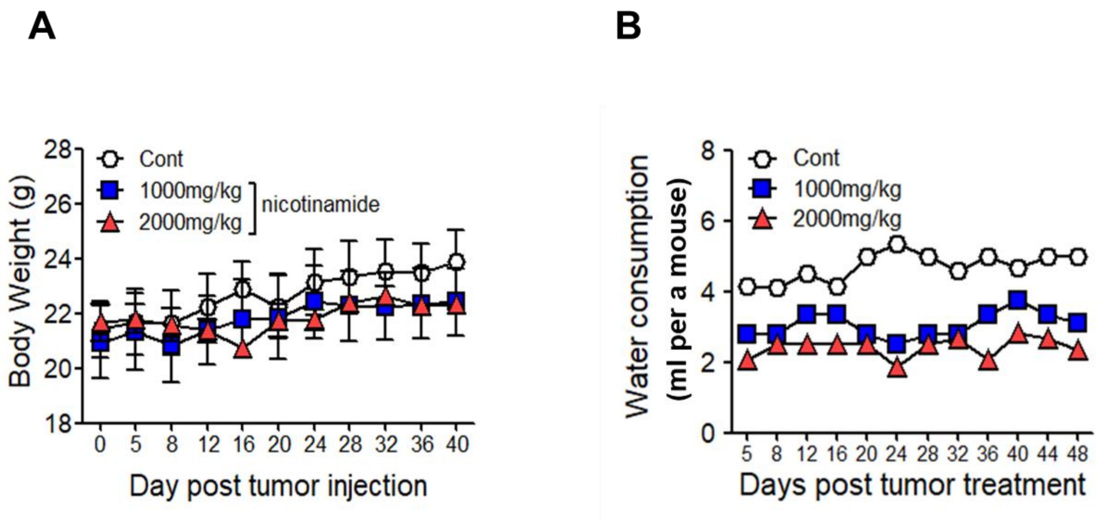

Fig. S3. Growth curves and voluntary water consumption in the animal model (A) Body weights were measured as indicated day post tumor injection. The graph shows the mean ± SD of body weight. (B) The amounts of water consumed per mouse of each group were measured during experiments.
