# Supplementary material for: Nicotinamide (niacin) supplement increases lipid metabolism and ROS‐induced energy disruption in triple‐negative breast cancer: potential for drug repositioning as an anti‐tumor agent
Source: Mol Oncol. 2022 Mar 25;16(9):1795–815. doi: 10.1002/1878-0261.13209 (PMC9067146; doi:10.1002/1878-0261.13209)
Supplement: Supplementary file 4 — Fig. S4. A network model for the decreased immune response in NAM‐treated TNBC cells. [file MOL2-16-1795-s006.pdf]

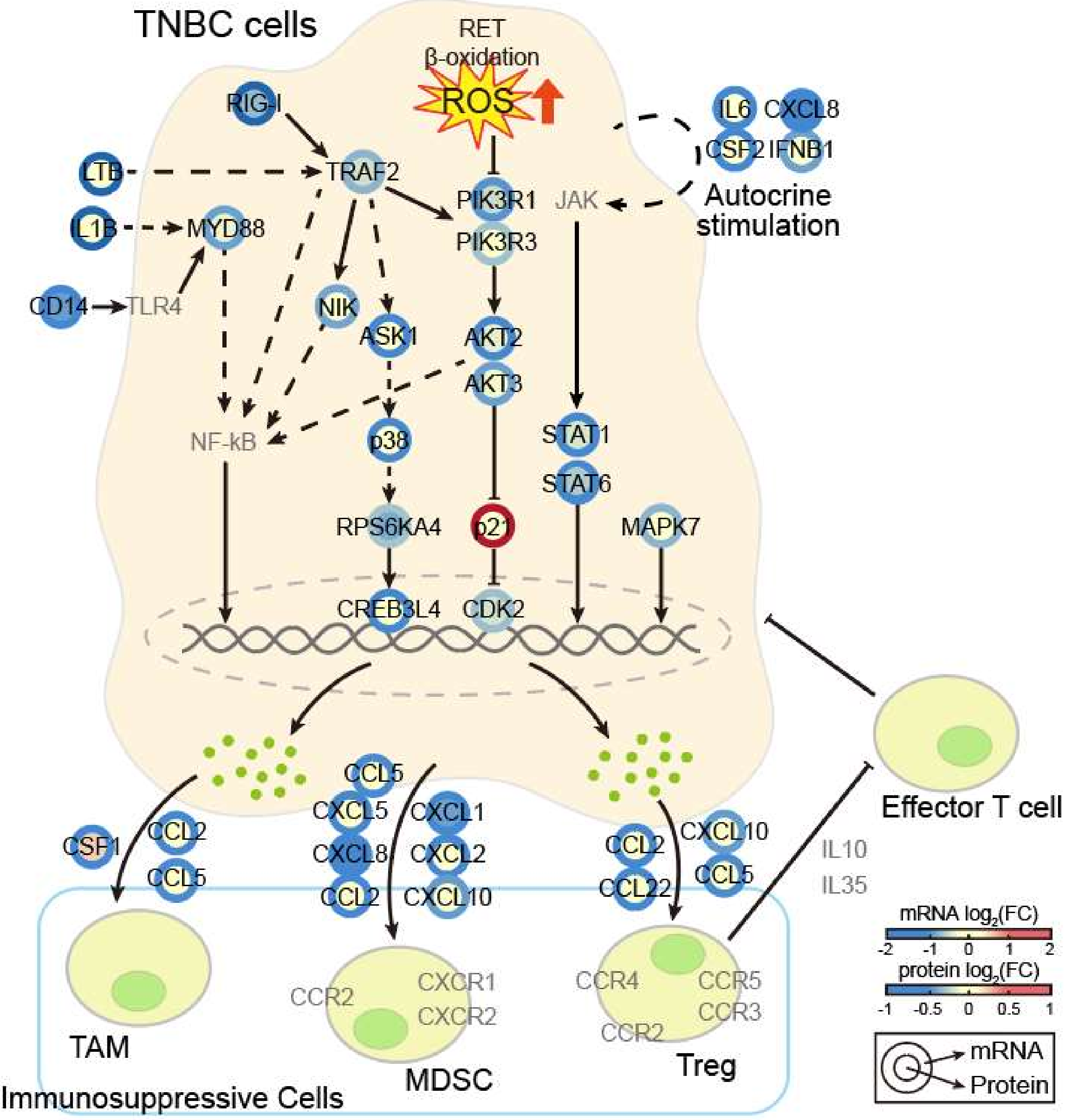

Cytokines	Targets	References	
**CCL2**	MDSC	Kumar V., et al. (2016). Trends in immunology, 37(3), 208–220.	[1]
	TAM	Hao, Q., et al. (2020). Cell communication and signaling: CCS, 18(1), 82.	[2]
	Treg	Chang, A. L., et al. (2016). Cancer research, 76(19), 5671–5682.	[3]
**CCL5**	MDSC	Kumar V., et al. (2016). Trends in immunology, 37(3), 208–220.	[1]
	TAM	Gao, D., et al. (2017) BMC cancer, 17(1), 834.	[4]
	Treg	Tan, M. C., et al. (2009). Journal of immunology (Baltimore, Md.: 1950), 182(3), 1746–1755	[5]

**CCL22**	Treg	Gobert, M., et al. (2009). Cancer research, 69(5), 2000–2009	[6]
**CSF1**	TAM	Ding, J., et al. (2016). International journal of oncology, 49(5), 2064–2074.	[7]
**CSF2**	Autocrine	Bhattacharya, P., et al. (2015). Cytokine, 75(2), 261–271.	[8]
**CXCL1**	MDSC	Shi, H., et al. (2018). Cancer science, 109(12), 3826–3839.	[9]
**CXCL2**	MDSC	Shi, H., et al. (2018). Cancer science, 109(12), 3826–3839.	[9]
**CXCL5**	MDSC	Zhang, W., et al. (2020). Cancer communications (London, England), 40(2-3), 69–80.	[10]
**CXCL8**	MDSC	Alfaro, C., et al. (2016). Clinical cancer research: an official journal of the American Association for Cancer Research, 22(15), 3924–3936	[11]
	Autocrine	Campbell, L. M., et al. (2013). Pharmaceuticals (Basel, Switzerland), 6(8), 929–959.	[12]
**CXCL10**	MDSC	Liu, H., et al. (2021). Cell death & disease, 12(5), 489.	[13]
	Treg	Li, C. X., et al. (2016). Journal of hepatology, 65(5), 944–952.	[14]
**IFNB1**	Autocrine	Yarilina, A., et al. (2008). Nature immunology, 9(4), 378–387.	[15]
**IL6**	Autocrine	Grivennikov, S., et al. (2008). Cancer cell, 13(1), 7–9.	[16]
**IL1B**	NF-kB signaling	Kawai, T., et al. (2007). Trends in molecular medicine, 13(11), 460–469.	[17]
**LTB**	NF-kB signaling	Chang, Y. H., et al. (2002). Experimental cell research, 278(2), 166–174.	[18]

Fig. S4. A network model for the decreased immune response in NAM-treated TNBC cells. Arrows indicate directions of the reactions, and dotted arrows indicate indirect reactions involving intermediate reactions between the nodes. Node colors represent up (red) and downregulation (blue) of the corresponding genes (node boundary) or proteins (node center). Color bar, gradient of log2-fold-changes in NAM versus control. The maximum of absolute log2-fold-changes in the three cell types were chosen for the node colors. Node label size denotes whether the corresponding gene was identified as a DEG in three (large), two (middle), or one (small) of the cell types. Grey node labels denote that the corresponding nodes are non-DEGs in the three cell types. Previous studies reporting actions of cytokines on their target cells indicated in the network model are summarized in the table.

Reference list

1 Kumar, V., Patel, S., Tcyganov, E., & Gabrilovich, D. I. (2016). The Nature of Myeloid-Derived Suppressor Cells in the Tumor Microenvironment. Trends in immunology, 37(3), 208–220.

2 Hao, Q., Vadgama, J. V., & Wang, P. (2020). CCL2/CCR2 signaling in cancer pathogenesis. Cell communication and signaling : CCS, 18(1), 82.

3 Chang, A. L., Miska, J., Wainwright, D. A., Dey, M., Rivetta, C. V., Yu, D., Kanojia, D., Pituch, K. C., Qiao, J., Pytel, P., Han, Y., Wu, M., Zhang, L., Horbinski, C. M., Ahmed, A. U., & Lesniak, M. S. (2016). CCL2 Produced by the Glioma Microenvironment Is Essential for the Recruitment of Regulatory T Cells and Myeloid-Derived Suppressor Cells. Cancer research, 76(19), 5671–5682.

4 Gao, D., Cazares, L. H., & Fish, E. N. (2017). CCL5-CCR5 interactions modulate metabolic events during tumor onset to promote tumorigenesis. BMC cancer, 17(1), 834.

5 Tan, M. C., Goedegebuure, P. S., Belt, B. A., Flaherty, B., Sankpal, N., Gillanders, W. E., Eberlein, T. J., Hsieh, C. S., & Linehan, D. C. (2009). Disruption of CCR5-dependent homing of regulatory T cells inhibits tumor growth in a murine model of pancreatic cancer. Journal of immunology (Baltimore, Md. : 1950), 182(3), 1746–1755.

6 Gobert, M., Treilleux, I., Bendriss-Vermare, N., Bachelot, T., Goddard-Leon, S., Arfi, V., Biota, C., Doffin, A. C., Durand, I., Olive, D., Perez, S., Pasqual, N., Faure, C., Ray-Coquard, I., Puisieux, A., Caux, C., Blay, J. Y., & Ménétrier-Caux, C. (2009). Regulatory T cells recruited through CCL22/CCR4 are selectively activated in lymphoid infiltrates surrounding primary breast tumors and lead to an adverse clinical outcome. Cancer research, 69(5), 2000–2009.

7 Ding, J., Guo, C., Hu, P., Chen, J., Liu, Q., Wu, X., Cao, Y., & Wu, J. (2016). CSF1 is involved in breast cancer progression through inducing monocyte differentiation and homing. International journal of oncology, 49(5), 2064–2074.

8 Bhattacharya, P., Thiruppathi, M., Elshabrawy, H. A., Alharshawi, K., Kumar, P., & Prabhakar, B. S. (2015). GM-CSF: An immune modulatory cytokine that can suppress autoimmunity. Cytokine, 75(2), 261–271.

9 Shi, H., Han, X., Sun, Y., Shang, C., Wei, M., Ba, X., & Zeng, X. (2018). Chemokine (C-X-C motif) ligand 1 and CXCL2 produced by tumor promote the generation of monocytic myeloid-derived suppressor cells. Cancer science, 109(12), 3826–3839.

10 Zhang, W., Wang, H., Sun, M., Deng, X., Wu, X., Ma, Y., Li, M., Shuoa, S. M., You, Q., & Miao, L. (2020). CXCL5/CXCR2 axis in tumor microenvironment as potential diagnostic biomarker and therapeutic target. Cancer communications (London, England), 40(2-3), 69–80.

11 Alfaro, C., Teijeira, A., Oñate, C., Pérez, G., Sanmamed, M. F., Andueza, M. P., Alignani, D., Labiano, S., Azpilikueta, A., Rodriguez-Paulete, A., Garasa, S., Fusco, J. P., Aznar, A., Inogés, S., De Pizzol, M., Allegretti, M., Medina-Echeverz, J., Berraondo, P., Perez-Gracia, J. L., & Melero, I. (2016). Tumor-Produced Interleukin-8 Attracts Human Myeloid-Derived Suppressor Cells and Elicits Extrusion of Neutrophil Extracellular Traps (NETs). Clinical cancer research : an official journal of the American Association for Cancer Research, 22(15), 3924–3936.

12 Campbell, L. M., Maxwell, P. J., & Waugh, D. J. (2013). Rationale and Means to Target Pro-Inflammatory Interleukin-8 (CXCL8) Signaling in Cancer. Pharmaceuticals (Basel, Switzerland), 6(8), 929–959.

13 Liu, H., Ling, C. C., Yeung, W., Pang, L., Liu, J., Zhou, J., Zhang, W. Y., Liu, X. B., Ng, T., Yang, X. X., Lo, C. M., & Man, K. (2021). Monocytic MDSC mobilization promotes tumor recurrence after liver transplantation via CXCL10/TLR4/MMP14 signaling. Cell death & disease, 12(5), 489.

14 Li, C. X., Ling, C. C., Shao, Y., Xu, A., Li, X. C., Ng, K. T., Liu, X. B., Ma, Y. Y., Qi, X., Liu, H., Liu, J., Yeung, O. W., Yang, X. X., Liu, Q. S., Lam, Y. F., Zhai, Y., Lo, C. M., & Man, K. (2016). CXCL10/CXCR3 signaling mobilized-regulatory T cells promote liver tumor recurrence after transplantation. Journal of hepatology, 65(5), 944–952.

15 Yarilina, A., Park-Min, K. H., Antoniv, T., Hu, X., & Ivashkiv, L. B. (2008). TNF activates an IRF1-dependent autocrine loop leading to sustained expression of chemokines and STAT1-dependent type I interferon-response genes. Nature immunology, 9(4), 378–387.

16 Grivennikov, S., & Karin, M. (2008). Autocrine IL-6 signaling: a key event in tumorigenesis?. Cancer cell, 13(1), 7–9.

17 Kawai, T., & Akira, S. (2007). Signaling to NF-kappaB by Toll-like receptors. Trends in molecular medicine, 13(11), 460–469.

18 Chang, Y. H., Hsieh, S. L., Chen, M. C., & Lin, W. W. (2002). Lymphotoxin beta receptor induces interleukin 8 gene expression via NF-kappaB and AP-1 activation. Experimental cell research, 278(2), 166–174.
