# Supplementary material for: Nicotinamide (niacin) supplement increases lipid metabolism and ROS‐induced energy disruption in triple‐negative breast cancer: potential for drug repositioning as an anti‐tumor agent
Source: Mol Oncol. 2022 Mar 25;16(9):1795–815. doi: 10.1002/1878-0261.13209 (PMC9067146; doi:10.1002/1878-0261.13209)
Supplement: Supplementary file 5 — Table S1. Primers used for qPCR assay. [file MOL2-16-1795-s007.pdf]

Table S1. Primers used for qPCR assay

Gene symbol	Forward	Reverse
SLC25A4	CATCGAGAGGGTCAAACTGC	CCCTCCAGAAGGAGAGGAAG
SLC1A5	GTGTTTGCCATCGTCTTTGG	GCGTACCACATGATCCAGG
SLC12A2	GATTTGCAGAAACCGTGGTG	TTTGCTTCCCACTCCATTCC
SLC2A1	TATGTGGAGCAACTGTGTGG	CGGCCTTTAGTCTCAGGAAC
SLC9A3R2	CGTCACCCGTCACCAATG	CTCAGTGTCCTTGTCGGAAC
SLC25A20	CCTGGAGAACGGATCAAGTG	GTCCCTTTGTAGATGCCTCG
SLC25A1	AAGGCATTCTACAAGGGCAC	AGTCCGTCTTCCACACTTTG
SLC4A7	TGTGGCAACTCTCTCTTTGC	GGCCAGAAGCTATCATGTTG
SLC44A1	TGCATTTGTTGCCTTTGGTG	TGGTGCTGTTGATAGCTGTG
SLC1A4	CTGTGGACTGGATTGTGGAC	CTGCTCGCCTTTCTTTGTTG
SLC7A11	CATGAACGGTGGTGTGTTTG	CTGGTAGAGGAGTGTGCTTG
SLC39A10	CTGGATTGACAGGAGGAATCA	CATGGCAGAGAGGAGGTTG
SLC7A2	TTGCCATCGTTCTCACCATC	AATCTGACCCAAGTGTCTGC
SLC38A1	TCTATGACAACGTGCAGTCC	ACAGCAACAATGACAGCCAG
SLC43A3	CTCTCAACTCCTTGCTGACC	TGTTTAAGCCGGTCCATGAG
SLC25A44	CAGCTCTCCTACCTGTGTCC	ATGATGGAGTTCTTGCCCTC
SLC38A2	TTAATGGCTGTGACCCTGAC	GAGACTATGACGCCACCAAC
SLC25A30	ACGCCCAGAAGATGAAACTC	TTGAATGGTGTTGCTTTGCG
SLC30A6	CCTTGATGACATTTGGCACT	GTCCAACTGACCAATAACATGG
SLC16A7	CTCCTTTAGTAGCAGCGTGG	TTATGGTTAAGGCGGGTTGC
SLC39A1	CGAGGCGGTAGCTTCAGAG	GAGCCTTCATGGTTAGCTCC
SLC25A23	GAGGAGCTGGACAGTAACAAG	AGAGGAGATACCCTGTTGGG
SLC35B1	TAGAAGAACACACAGTCGGC	CTGGGAAACACCAGTCAGTC
SLC12A4	CTTCCCTCGTGTACTTCAGC	TGACACCATCGCCATACTTG
SLC29A1	AGCAGGCAAAGAGGAATCTG	CAGACAGAGAAAGCCAGGAC
SLC1A3	AGTCCGTAAACGCACACTTT	TCAAATGACTCACCCACAATG
SLC5A1	TTGCCATTATCCTCTTCGCC	CACAGACGGTAGAGATGCAC
SLC6A14	CTATGGTGGAGAGCTTGCTG	AGTCAGGGTATGGAATTGCG
SLC4A11	CTGACAACAATGAGCCCAAC	TGACAGCAGGTGGACTTTAC
SLC35A1	GTGCCATCGTTAGTGTATGC	ACGGAATCTTCAACTGGTAGG
SLC36A1	GGTGAAATGTGCTCACCACT	AGGAAGAAGTCCACAACACG
SLC25A52	CTACCGCAACGACTCACAG	CAAGAATCCCAACATGGCAC

Gene symbol	Forward	Reverse
SLC3A2	GAACTCGTGGTTCTCCACTC	TCAGCCAAGAATGAGGATGC
SLC33A1	TTGTCGAACACCTGATGCTG	ACCAAGAAAGAACCACCAACC
SLC7A5	CGTGGACTTCGGGAACTATC	AAGAGCCTGGAGGATGTGAA
SLC39A14	CGACGGCCTCCATAATTTCA	AGTCTCCTAGCTCATGTGGG
SLC7A1	TAATCGCCACATTAGCCTCG	GTAAGACCAACACACAGGCA
SLC25A25	GAGAGACTACCACCTCCTCC	TCTCACCCACATCAAAGATCG
SLC16A3	CAGTTCGAGGTGCTCATGG	ATGTAGACGTGGGTCGCATC
RPL13A	GTGTTTGACGGCATCCCACC	TAGGCTTCAGACGCACGACC
ACSL3	CCGGCGTAGCGATACAGAAT	AGCTTCTGAGGGTGGCAAAT
CPT2	GCTGGCCAGGGCTTTGA	AGGGTCCAGGTAGAGCTCAG
CPT1A	GTTCTCTTGCCCTGAGACGG	TTTCCAGCCCAGCACATGAA
HADHB	CCCACTGCATCAAAATGGGC	CAACAAACCCGAAGTGCCAG
ETFDH	GCACCAAAGGCAACATTTGAG	AGTCCAATCCCGTAGGTTTGA
