# Supplementary material for: Nicotinamide (niacin) supplement increases lipid metabolism and ROS‐induced energy disruption in triple‐negative breast cancer: potential for drug repositioning as an anti‐tumor agent
Source: Mol Oncol. 2022 Mar 25;16(9):1795–815. doi: 10.1002/1878-0261.13209 (PMC9067146; doi:10.1002/1878-0261.13209)
Supplement: Supplementary file 8 — Table S4. The IC50 value of NAM treatment to TNBC cell lines. [file MOL2-16-1795-s001.pdf]

Table S4. The IC_{50} value of NAM treatment to TNBC cell lines

**Cell lines**	**IC_{50} (mM)**
BT20	21.4 ± 1.42
MDA-MB-468	27.9 ± 1.40
MDA-MB-231	34.7 ± 3.89
