# Supplementary material for: Nicotinamide (niacin) supplement increases lipid metabolism and ROS‐induced energy disruption in triple‐negative breast cancer: potential for drug repositioning as an anti‐tumor agent
Source: Mol Oncol. 2022 Mar 25;16(9):1795–815. doi: 10.1002/1878-0261.13209 (PMC9067146; doi:10.1002/1878-0261.13209)
Supplement: Supplementary file 9 — Table S5. The IC50 value of NAM treatment to TNBC organoids. [file MOL2-16-1795-s004.pdf]

Table S5. The IC_{50} value of NAM treatment to TNBC organoids

**TNBC organoids**	**IC_{50} (mM)**
Patient #1	18.0 ± 0.89
Patient #2	12.8 ± 0.39
Patient #3	29.1 ± 1.77
Patient #4	29.9 ± 1.48
